# Pyrroloquinoline quinone promotes human mesenchymal stem cell-derived mitochondria to improve premature ovarian insufficiency in mice through the SIRT1/ATM/p53 pathway

**DOI:** 10.1186/s13287-024-03705-4

**Published:** 2024-04-05

**Authors:** Shengjie Liu, Yuanmei Wang, Hanlin Yang, Jun Tan, Jingkaiwen Zhang, Dan Zi

**Affiliations:** 1 GuiZhou University Medical College, Guiyang, Guizhou Province 550025 China; 2 Department of Gynaecology and Obstetrics, The Affiliated Hospital of Guizhou Medical University, Guizhou Medical University, Guiyang, 550004 China; 3 Department of Gynecology, Guizhou Provincial People’s Hospital, Guiyang, Guizhou 550025 China; 4 Key Laboratory of Endemic and Ethnic Diseases and Key Laboratory of Molecular Biology, Ministry of Education, Guizhou Medical University, Guiyang, 550004 China

**Keywords:** Premature ovarian insufficiency, DNA damage, Oxidative stress, Mesenchymal stem cells, Pyrroloquinoline quinone, Mitochondrial transplantation

## Abstract

**Background:**

DNA damage and oxidative stress induced by chemotherapy are important factors in the onset of premature ovarian insufficiency (POI). Studies have shown that mitochondria derived from mesenchymal stem cells (MSC-Mito) are beneficial for age-related diseases, but their efficacy alone is limited. Pyrroloquinoline quinone (PQQ) is a potent antioxidant with significant antiaging and fertility enhancement effects. This study aimed to investigate the therapeutic effect of MSC-Mito in combination with PQQ on POI and the underlying mechanisms involved.

**Methods:**

A POI animal model was established in C57BL/6J mice by cyclophosphamide and busulfan. The effects of MSC-Mito and PQQ administration on the estrous cycle, ovarian pathological damage, sex hormone secretion, and oxidative stress in mice were evaluated using methods such as vaginal smears and ELISAs. Western blotting and immunohistochemistry were used to assess the expression of SIRT1, PGC-1α, and ATM/p53 pathway proteins in ovarian tissues. A cell model was constructed using KGN cells treated with phosphoramide mustard to investigate DNA damage and apoptosis through comet assays and flow cytometry. SIRT1 siRNA was transfected into KGN cells to further explore the role of the SIRT1/ATM/p53 pathway in combination therapy with MSC-Mito and PQQ for POI.

**Results:**

The combined treatment of MSC-Mito and PQQ significantly restored ovarian function and antioxidant capacity in mice with POI. This treatment also reduced the loss of follicles at various stages, improving the disrupted estrous cycle. In vitro experiments demonstrated that PQQ facilitated the proliferation of MitoTracker-labelled MSC-Mito, synergistically restoring mitochondrial function and inhibiting oxidative stress in combination with MSC-Mito. Both in vivo and in vitro, the combination of MSC-Mito and PQQ increased mitochondrial biogenesis mediated by SIRT1 and PGC-1α while inhibiting the activation of ATM and p53, consequently reducing DNA damage-mediated cell apoptosis. Furthermore, pretreatment of KGN cells with SIRT1 siRNA reversed nearly all the aforementioned changes induced by the combined treatment.

**Conclusions:**

Our research findings indicate that PQQ facilitates MSC-Mito proliferation and, in combination with MSC-Mito, ameliorates chemotherapy-induced POI through the SIRT1/ATM/p53 signaling pathway.

**Supplementary Information:**

The online version contains supplementary material available at 10.1186/s13287-024-03705-4.

## Introduction

Premature ovarian insufficiency (POI) refers to the decline in or complete loss of ovarian function in women before the age of 40 [1]. This condition is characterized by depletion of the primordial follicle pool, which leads to ovarian dysfunction [2]. Its hallmark features include irregular or absent menstruation, disrupted oestradiol (E2) levels, and elevated follicle-stimulating hormone (FSH) levels [3]. Currently, hormone replacement therapy and ovarian tissue transplantation can alleviate some symptoms of POI, but their therapeutic effects are temporary and cannot effectively repair self-inflicted damage to ovarian tissue [4]. Therefore, there is an urgent need to explore safe and effective treatment modalities.

Chemotherapy is a major cause of iatrogenic POI. Research has indicated that approximately one-third of women diagnosed with cancer under the age of 40 experience POI after chemotherapy [5–7]. Cyclophosphamide is a commonly used chemotherapy drug, and its detrimental effects on the ovaries include the excessive activation of primordial follicles, depletion of ovarian reserves, and its active metabolite, Phosphoramide mustard cyclohexanamine (PM), crosslinking to DNA from egg granulosa cells and oocytes, inhibiting their synthesis and even causing DNA double-strand breaks, leading to apoptosis [8–10]. Studies have shown that cyclophosphamide is involved in the development of POI through various mechanisms, including elevated oxidative stress, intense inflammatory responses, disruption of DNA damage repair pathways, and mitochondrial damage.

As cellular powerhouses, mitochondria play pivotal roles in various physiological functions. Their dysfunction is considered one of the contributing factors to the occurrence of POI. The substantial production of reactive oxygen species (ROS) resulting from mitochondrial dysfunction is closely associated with follicular atresia and ovarian ageing [11–13]. Previous studies have suggested that the use of stem cells for intercellular mitochondrial transfer can enhance cellular vitality, suggesting a potential therapeutic approach for treating mitochondrial dysfunction-related disorders [14]. However, this therapeutic approach has limitations, including a lower survival rate of transplanted stem cells and reduced efficiency in intercellular mitochondrial transfer. Moreover, concerns related to immune rejection, tumorigenicity, and numerous ethical issues have been raised. In contrast, the transplantation of mitochondria directly into damaged tissues for tissue repair is considered a highly attractive therapeutic approach. Importantly, direct mitochondrial transplantation prevents immune rejection and helps mitigate ethical concerns. A wealth of evidence suggests that mitochondrial transplantation has favourable outcomes in the treatment of neurological disorders such as Alzheimer’s disease and cardiovascular conditions such as myocardial ischaemia‒reperfusion injury [15–17]. Our previous research revealed that liver-derived mitochondrial transplantation could improve sex hormone levels and increase the number of various stages of ovarian follicles in mice with POI. This result laid the theoretical foundation for the current study. Obtaining a substantial quantity of high-quality autologous mitochondria from one’s own body is a challenging endeavour. Consequently, stem cells have emerged as a prevalent source of mitochondria. Among these stem cells, mesenchymal stem cells, characterized by their low differentiation state and ease of cultivation, have become a commonly utilized source of mitochondria. While some evidence suggests that mitochondrial transplantation contributes to recovery from certain diseases, the efficiency of mitochondrial transfer is not particularly high. This inefficiency has impacted the practical application and clinical translation of mitochondrial therapies [18]. The presence of a substantial quantity of free radicals in the ovarian cells of patients with POI is likely a contributing factor affecting the success of mitochondrial transplantation. The diminished capacity to clear reactive oxygen species is intricately linked with ovarian ageing, and several studies have indicated that the use of antioxidants can decelerate ovarian ageing.

Pyrroloquinoline quinone (PQQ), as a novel redox cofactor, functions as a highly efficient free radical scavenger, possessing potent antiaging and antioxidant properties that play a crucial role in maintaining cellular redox balance. Research has indicated that PQQ can promote mitochondrial biogenesis and enhance cellular antiageing capabilities [19]. Furthermore, PQQ can reduce ROS levels, decrease granulosa cell apoptosis, facilitate follicular development, and benefit female reproduction while mitigating cyclophosphamide-induced ovarian damage [20, 21].

Sirtuin 1 (SIRT1) is an NAD-dependent deacetylase known as an antiageing factor. SIRT1 plays a pivotal role in regulating various physiological functions, including the cell cycle, DNA damage response, energy metabolism, cell ageing, and apoptosis [22, 23]. The SIRT1 protein can modulate cell proliferation or apoptosis by deacetylating downstream substrates such as p53 and FOXO1 [24, 25]. Studies have shown that the loss of SIRT1 increases ATM protein expression in DNA damage models, leading to the activation of p53-mediated cell cycle arrest and apoptosis [26]. SIRT1 can also activate PPARγ coactivator-1 alpha (PGC-1α) to regulate mitochondrial biogenesis [27, 28]. Numerous studies have confirmed the strong association between SIRT1 and the proliferation, apoptosis, oocyte quality, follicular maturation, and ageing of ovarian granulosa cells [29–31]. This finding indicates the pivotal regulatory role of SIRT1 in ovarian function and renders it a potential target for addressing POI. Investigations into SIRT1 and its regulatory mechanisms hold promise for introducing novel perspectives and methodologies in the treatment of POI.

To date, there have been no reports on the combined treatment of POI involving mitochondrial transplantation and PQQ. Previous research has indicated the beneficial effects of antioxidative measures and enhanced mitochondrial function in addressing POI. However, standalone interventions using mitochondrial transplantation or PQQ have failed to fully restore ovarian function in chemotherapy-induced POI. Therefore, this study focuses on whether PQQ can augment mitochondrial transplantation in the treatment of chemotherapy-induced POI and explores the underlying molecular mechanisms at play.

## Materials and methods

### Animals

Six-week-old female SPF-grade C57BL/6J mice were obtained from SpePharm (Beijing) Biotechnology Co., Ltd. All mice were housed at a temperature of 20–26 °C with a relative humidity of 40-70%. The animals were subjected to a 12-hour light/dark cycle and had ad libitum access to food and water. Experimental procedures commenced one week after acclimatization to the housing conditions. The use of animals in this study was approved by the Ethics Committee for Animal Experiments at Guizhou University.

### Isolation and functional assessment of mitochondria

Mitochondria were isolated from 4th to 7th passage human umbilical cord mesenchymal stem cells (KarlCell Bio, China) using a cell mitochondrial extraction kit (Thermo, USA). The structural examination of mitochondria was conducted with transmission electron microscopy. Mitochondrial membrane potential was evaluated using MitoTracker® Red FM staining solution (Thermo, USA) and the JC-1 mitochondrial membrane potential detection kit (Beyotime, China). The mitochondrial concentration was determined using the Coomassie Brilliant Blue G250 assay (Solarbio, China).

### Establishment and treatment of POI model mice

Thirty-five mice were randomly divided into five groups: control, POI, POI + PQQ, POI + Mito, and POI + PQQ + Mito. Except for those in the control group, all mice were induced to establish the POI animal model by a single intraperitoneal injection of 60 mg/kg cyclophosphamide (Sigma, USA) and 15 mg/kg busulfan (Solarbio, China). In the PQQ treatment group, the mice were orally administered 15 mg/kg PQQ (Sigma, USA) daily for three weeks. In the mitochondrial treatment group, each mouse received 200 µg of mitochondria via intraperitoneal injection twice a week for three weeks. In the combined treatment group, the mice were orally administered 15 mg/kg PQQ daily and received intraperitoneal injections of 200 µg mitochondria twice a week for three weeks. The control group and the POI group received equivalent volumes of normal saline via oral gavage and intraperitoneal injection at the same time. Two days before the end of the experiment, each mouse was subcutaneously injected with 0.1 ml of pregnant mare serum gonadotropin (PMSG) (Solarbio, China) to synchronize their oestrous cycles. Twelve hours before euthanasia, food and water were withheld. Mice were anesthetized by intraperitoneal injection of 50 mg/kg sodium pentobarbital. Blood is collected by removing the eyeball, and then the skin and muscle layers are cut from the abdomen to extract bilateral ovarian tissue. Collected blood samples were allowed to stand at room temperature for 1 h, followed by centrifugation at 3000 rpm at 4 °C for 10 min. The upper serum layer was separated, sealed, and stored at -20 °C for later use. One side of the collected ovarian tissue was fixed in 10% neutral formalin and the other side was stored in an ultra-low temperature refrigerator at -80 ℃. The work has been reported in line with the ARRIVE 2.0 guidelines.

### Vaginal smear

Vaginal smear examinations were conducted daily at 10:30 AM to observe the oestrous cycles in each group. A cotton swab for children moistened with double-distilled water was gently inserted into the mouse’s vagina and rotated clockwise 3–4 times. The collected vaginal cells were evenly spread on a glass slide. After the smear was naturally air-dried, the cells were fixed using wright stain solution (Solarbio, China). Changes in the oestrous cycle were observed under a microscope.

### Enzyme-linked immunosorbent assay (ELISA)

An ELISA kit (Jingmei, China) was utilized following the manufacturer’s instructions to measure the levels of anti-Müllerian hormone (AMH), oestradiol (E2), and follicle-stimulating hormone (FSH) in the serum of mice from each group. In brief, 50 µL of 5-fold diluted serum samples was added to the wells of an enzyme-coated plate. After incubation with the enzyme-conjugated reagent, the plate was washed five times. Subsequently, a mixture of chromogenic reagents A and B was added, followed by the addition of 50 µL stop solution. The absorbance at 450 nm was measured for each well after zeroing against blank wells.

### Follicle counting

After three weeks of treatment with pyrroloquinoline quinone and mitochondria, the ovaries of each mouse were collected. The ovaries were fixed in 4% paraformaldehyde, embedded in paraffin, and sectioned. Haematoxylin and eosin (H&E) staining was performed on the sections. Based on the distinct morphological characteristics of follicles, the primordial follicles, primary follicles, secondary follicles, mature follicles, and atretic follicles were counted for each slide. Subsequently, the proportions of different follicle types in each group were analysed.

### Immunohistochemistry (IHC)

The paraffin-embedded ovarian tissue sections from each group of mice were deparaffinized by baking in a constant temperature oven at 60 °C for 1 h. Subsequently, the samples were deparaffinized and hydrated using xylene and a gradient of ethanol. After high-pressure antigen retrieval with citrate repair solution and removal of endogenous peroxidase, the sections were blocked using 5% specialized goat serum. They were then incubated overnight at 4 °C with the appropriate primary antibodies. Subsequently, incubation with goat anti-rabbit secondary antibodies was carried out at 37 °C for 1 h. DAB staining solution was applied for 5–10 min, followed by counterstaining with haematoxylin for 20 s. After dehydration and sealing the slides, the sections were observed and recorded under a microscope.

### Cell culture and grouping

KGN cells were obtained from Procell Life Science Technology Co., Ltd., for in vitro research. KGN cells were cultured in DMEM/F12 medium containing 10% foetal bovine serum. The culture conditions were maintained at 37 °C with a 5% CO_2_ atmosphere. For establishment of an in vitro model of POI, the active metabolite of cyclophosphamide, phosphoramide mustard (PM) (MedChemExpress, China), was used. The cells were divided into 5 groups: the normal control group (control), the model group (PM), the pyrroloquinoline quinone treatment group (PM + PQQ), the mitochondria treatment group (PM + Mito), and the combination treatment group (PM + PQQ + Mito).

### Cell proliferation assay (CCK-8)

KGN cells were seeded in a 96-well plate at a density of 1×10^4^ cells/mL, with 100 µL of cell suspension in each well. Each group had at least 3 replicate wells. The plate was placed in a 37 °C, 5% CO_2_ incubator until the cells reached approximately 80% confluence. The culture medium was then aspirated, and different concentrations of PM, Mito, and PQQ were added to the wells. The cells were incubated in the incubator for an appropriate amount of time. Next, 10 µL of CCK-8 enhanced solution (Meilunbio, China) was added to each well, and the plate was further incubated in the incubator for 0.5 h. The optical density (OD) at 450 nm was measured.

### Measurement of mitochondrial membrane potential

The mitochondrial membrane potential was determined using the JC-1 mitochondrial membrane potential assay kit. To each well of a six-well plate, 1 mL of JC-1 staining working solution and 1 mL of cell culture medium were added. The mixture was thoroughly mixed and then placed in a cell culture incubator at 37 °C for 20 min. Afterwards, the cells were washed twice with JC-1 staining buffer (1×) and observed under a fluorescence microscope.

### Real-time fluorescence quantitative PCR (RT‒qPCR)

Total DNA from cells in each group was extracted using a DNA extraction kit (Tiangen Biotech, China). Total RNA from processed KGN cells was extracted using TRIzol™ reagent (Thermo, USA). Subsequently, the RNA was reverse transcribed into cDNA using a reverse transcription kit (TaKaRa, Japan). PCR amplification of the target genes was performed using the TB Green PCR reagent kit (TaKaRa, Japan). The relative expression levels of the target genes were determined using the 2^-ΔΔCt^ method, with GAPDH as the negative control. The primer sequences are provided in Table 1.

**Table 1:** List of primers in RT-qPCR

Species	Gene	Direction	Sequence
Human	ND1	Forward	GGAGTAATCCAGGTCGGT
		Reverse	TGGGTACAATGAGGAGTAGG
	SIRT1	Forward	GCCTCACATGCAAGCTCTAGTGAC
		Reverse	TTCGAGGATCTGTGCCAATCATAA
	PGC-1α	Forward	GCTGACAGATGGAGACGTGA
		Reverse	TAGCTGAGTGTTGGCTGGTG
	GAPDH	Forward	CAGAACATCATCCCTGCCTCTAC
		Reverse	TTGAAGTCAGAGGAGACCACCTG
Mouse	IL1α	Forward	TGGTTAAATGACCTGCAACAGGAA
		Reverse	AGGTCGGTCTCACTACCTGTGATG
	IL1β	Forward	TCCAGGATGAGGACATGAGCAC
		Reverse	GAACGTCACACACCAGCAGGTTA
	IL6	Forward	GTTCTCTGGGAAATCGTGGA
		Reverse	GGAAATTGGGGTAGGAAGGA
	TNFα	Forward	TATGGCCCAGACCCTCACA
		Reverse	GGAGTAGACAAGGTACAACCCATC
	GAPDH	Forward	TGAAGCAGGCATCTGAGGG
		Reverse	CGAAGGTGGAAGAGTGGGAG

### ROS measurement

DCFH-DA (Solarbio, China) was diluted in serum-free culture medium at a 1:1000 ratio, resulting in a final concentration of 10 µmol/L. After the cells were collected, they were suspended in diluted DCFH-DA solution, achieving a cell concentration of one to twenty million cells/mL. The cells were then incubated at 37 °C in a cell culture incubator for 20 min. Every 3–5 min, the solution was gently mixed to ensure proper contact between the probe and the cells. Afterwards, the cells were washed three times with serum-free cell culture medium to remove any remaining DCFH-DA that had not entered the cells. The fluorescence intensity was measured using a fluorescence microplate reader at an excitation wavelength of 488 nm and an emission wavelength of 525 nm.

### SOD and MDA measurement

Mouse ovarian tissues were retrieved from a -80 °C freezer and weighed accurately. The tissues were then homogenized by adding 9 times the volume of physiological saline based on tissue weight (g): volume (mL) = 1:9 ratio. After tissue homogenization, the mixture was centrifuged at 2500–3000 rpm for 10 min, and the supernatant, which was the 10% homogenate, was collected. The SOD and MDA levels were measured according to the instructions provided by the manufacturer (Nanjing Jiancheng Bioengineering Institute, China).

### Flow cytometry

Apoptosis levels in various cell groups were assessed using the Cell Apoptosis Detection Kit (BD Biosciences, USA). Cells were digested with trypsin, washed twice with prechilled PBS, and then resuspended in 1× binding buffer at a concentration of 1 × 10^6^ cells/mL. A total of 100 µL of the suspension (1 × 10^5^ cells) was transferred to a 5 mL culture tube. To this, 3 µL of FITC Annexin V and 5 µL of PI were added. The cells were gently vortexed and incubated in the dark at room temperature (25 °C) for 15 min. Subsequently, 300–400 µL of 1× binding buffer was added to each tube, and flow cytometry analysis was conducted within 1 h.

### Comet assay

Drug-treated KGN cells were collected in a 1.5 mL centrifuge tube. Ninety microlitres of 0.8% low-melting-point agarose gel was mixed with 10 µL of cell suspension containing approximately 1000 cells. Then, this mixture was spread onto a 1% normal-melting-point agarose gel quickly, covered with a coverslip, ensuring that there were no air bubbles, and placed in a 4 °C refrigerator for 10 min to solidify the gel. The glass slide was placed into the prechilled cell lysis working solution and incubated at 4 °C for 1 h. The glass slide was submerged horizontally into the prechilled alkaline electrophoresis working solution, avoiding light, and samples were incubated for 20 min. Then, the glass slide was placed into an electrophoresis chamber, and prechilled alkaline electrophoresis buffer was poured in, ensuring that the liquid level was approximately 0.25 cm above the glass slide. Electrophoresis was performed for 30 min at 25 V. Subsequently, the glass slide was placed in neutralization buffer for three cycles, each lasting 10 min. Then, 30 µL of propidium iodide (PI) staining solution was added to each glass slide, and the sample was covered with a coverslip and stained for 10 min, avoiding light. Finally, the cells were observed and images were captured under a fluorescence microscope.

### siRNA transfection

SIRT1-siRNA was designed and synthesized by Sangon Biotech. The SIRT1-siRNA sequences were as follows

Forward: 5’-GCGGGAAUCCAAAGGAUAAUUTT-3’.

Reverse: 5’-AAUUAUCCUUUGGAUUCCCGCTT-3’

Control-siRNA

Forward: 5’-UUCUCCGAACGUGUCACGUTT-3’

Reverse: 5’-ACGUGACACGUUCGGAGAATT-3’.

KGN cells were cultured in a 6-well plate until they reached 70-80% confluence. Then, siRNA, serum-free culture medium, and transfection reagent Lipo8000™ were diluted in the appropriate proportions. The siRNA mixture was added to the cells in the 6-well plate. The cells were transfected for two days, and then SIRT1 protein expression was assessed.

### Western blot analysis

Murine ovarian tissue and drug-treated KGN cells were subjected to protein extraction in RIPA lysis and extraction buffer (Thermo, USA). Protein concentrations were quantified using the BCA protein assay kit (Thermo, USA). The protein samples were fractionated by SDS‒PAGE and then transferred to PVDF membranes (Millipore, USA). Subsequently, the membrane was blocked using 5% skim milk for 1 h. Then, the sections were incubated overnight at 4 °C with the corresponding primary antibodies, including pATM, PGC-1α, TERF2 (ABclonal, China), SIRT1, p53 (Proteintech Group, China), Bax, Bcl-2, and β-actin (HuaBio, China), and then probed with secondary antibodies. Protein chemiluminescence was detected using the ECL chemiluminescence reagent (Thermo, USA). Finally, exposure was performed using an exposure device.

### Statistical analysis

The results are presented as the mean ± SEM. Statistical analysis was carried out using GraphPad Prism version 9.0 software, with data analysed using one-way analysis of variance (ANOVA) or independent sample t tests, as appropriate. Each group of samples underwent a minimum of three replicates, and statistical significance was defined as a *P* value < 0.05.

## Results

### The functional identification of MSC-Mito

Mitochondria were stained using the far-red fluorescent dye MitoTracker® Red FM, whose fluorescence intensity increases with activity. As depicted in Fig. S1A, the extracted mitochondria displayed strong red fluorescence, indicating that the free mitochondria extracted from mesenchymal stem cells were highly active. The assessment of mitochondrial membrane potential was conducted through the JC-1 assay. As depicted in Fig. S1B, the extracted mitochondria displayed robust red fluorescence while exhibiting subdued green fluorescence. However, upon the addition of the mitochondrial oxidative phosphorylation uncoupler CCCP, green fluorescence increased, indicating that the extracted mitochondria had a high membrane potential. We detected a high level of ATP in the extracted mitochondria, which significantly decreased after treatment with CCCP, confirming the excellent functionality of the extracted mitochondria (Fig. S1C). Transmission electron microscopy images of mitochondria revealed a round or oval shape, intact outer membranes, and well-defined cristae, indicating that the extracted mitochondria possessed intact structures (Fig. S1D).

### Synergistic improvement of ovarian function in mice with POI by MSC-Mito and PQQ

**Fig. 1:**
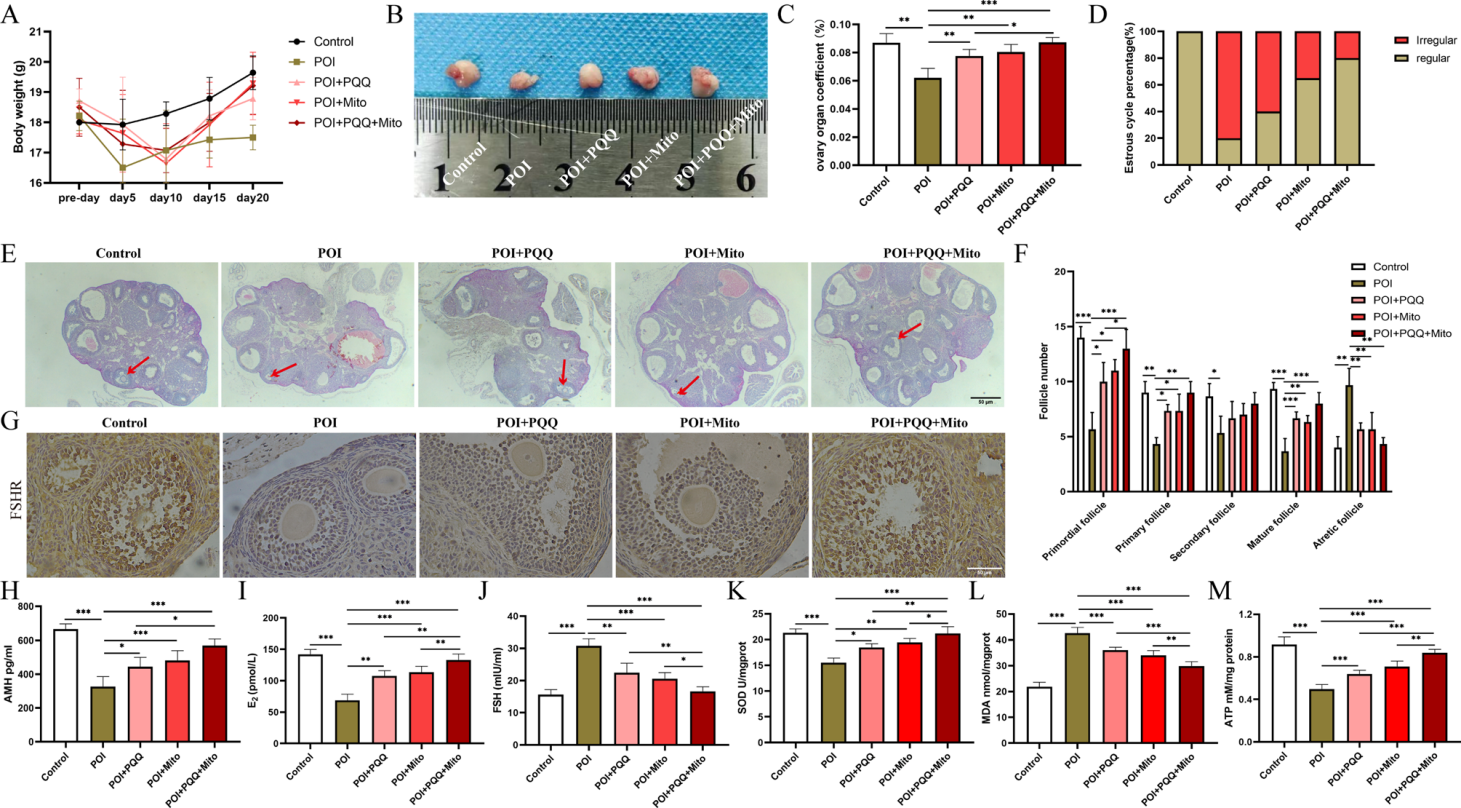
Synergistic improvement of ovarian function in mice with POI by MSC-Mito and PQQ. (**A**) Changes in the body weight of mice in each group. (**B**) Ovarian diameter and morphology of the mice in each group. (**C**) Ovarian organ index of the mice in each group. (**D**) Proportion of abnormal oestrous cycles in the mice in each group. (**E**) Histological staining of ovarian tissue sections using HE. Red arrows indicate mature follicles. (**F**) Count of follicles at different stages and atretic follicles. (**G**) IHC assessment of FSHR receptor levels in granulosa cells of the ovaries from different groups of mice. (**H-J**) ELISA analysis of serum levels of AMH, E2, and FSH hormones in various groups of mice. (**K-L**) Measurement of SOD and MDA levels in ovarian tissues of different groups of mice. (**M**) Measurement of ATP content in ovarian tissues from different groups of mice. Scale bar: 50 μm. Values are the means ± SEMs. *P < 0.05, **P < 0.01, ***P < 0.001

To assess the impact of MSC-Mito and PQQ on ovarian function in mice with POI, we observed changes in body weight, ovarian organ index, oestrous cycle, ovarian histopathology, sex hormone secretion, and antioxidant capacity among the groups. Compared to the control mice, the mice with POI exhibited significant reductions in body weight, ovarian volume, and ovarian relative weight. Nevertheless, the combined treatment of MSC-Mito and PQQ progressively restored the body weight and ovarian relative weight of the mice and substantially increased the ovarian volume, which approached the values of the control group (Fig. 1A-C). Vaginal smear analysis in mice revealed a gradual restoration of the oestrous cycle in mice with POI following combined treatment with MSC-Mito and PQQ (Fig. 1D). Haematoxylin and eosin staining revealed that, compared to the control group, the POI group exhibited a decrease in various follicle stages, an increase in atretic follicles, and notable ovarian interstitial fibrosis. Treatment with MSC-Mito and PQQ significantly increased the numbers of primordial, primary, and mature follicles while reducing the production of atretic follicles (Fig. 1E-F). Immunohistochemical analysis was employed to assess the expression of the granulosa cell marker FSHR receptor in the ovaries. The combined treatment of MSC-Mito and PQQ significantly mitigated the decrease in FSHR receptor expression, leading to an augmentation in the quantity of ovarian granulosa cells. (Fig. 1G). The assessment of sex hormone secretion in mice was conducted via ELISAs. We observed that in comparison to the control group, the POI group exhibited a significant decrease in AMH and E2 hormone levels, while FSH hormone levels markedly increased. However, the combined treatment with MSC-Mito and PQQ facilitated the restoration of sex hormone secretion, resulting in a substantial elevation in AMH and E2 hormone levels and a decline in FSH hormone levels, effectively approaching normal levels (Fig. 1H-J). We subsequently assessed the antioxidant capacity by measuring the levels of SOD and MDA in mouse ovaries. The results demonstrated that the combined treatment of MSC-Mito and PQQ significantly increased SOD levels and decreased MDA levels, indicating a marked enhancement in ovarian antioxidant capacity (Fig. 1K-L). We also assessed the ATP levels in mouse ovarian tissues and found a significant decrease in ATP content in the POI group compared to the control group. However, treatment with MSC-Mito and PQQ restored mitochondrial capacity (Fig. 1M). Furthermore, we observed that MSC-Mito and PQQ significantly reduced the gene expression levels of the inflammatory factors IL1α, IL1β, IL6, and TNFα in the ovaries of mice with POI, thereby suppressing the inflammatory response (Fig. S3A-D).

### PQQ facilitates MSC-Mito proliferation and synergistically MSC-Mito restores mitochondrial function and suppresses oxidative stress

**Fig. 2:**
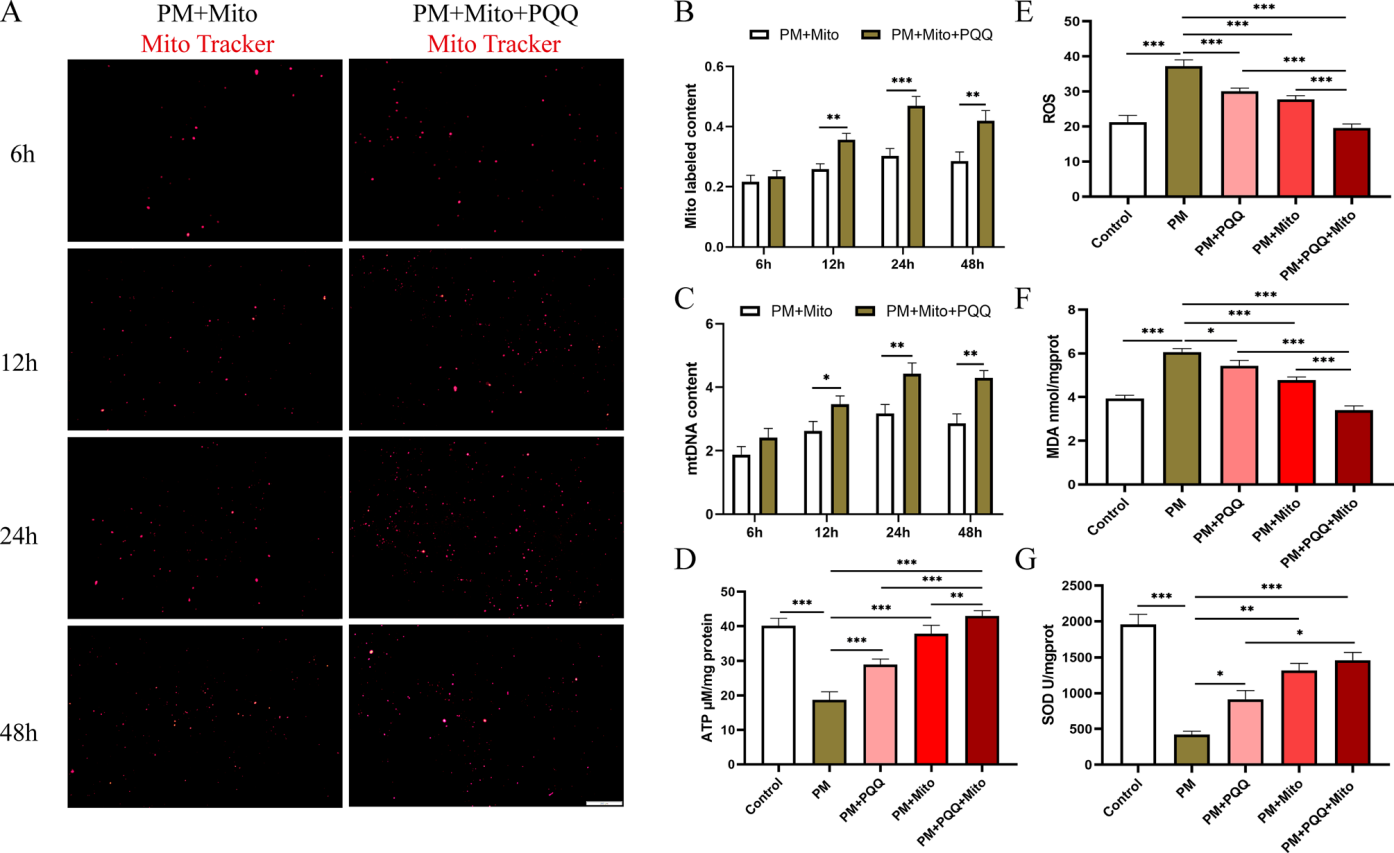
PQQ facilitates MSC-Mito proliferation and synergistically MSC-Mito restores mitochondrial function and suppresses oxidative stress. (**A**) Observation of the quantity of MitoTracker-labelled MSC-Mito in the cells using fluorescence microscopy. (**B**) Quantitative analysis of MitoTracker-stained MSC-Mito in the cells. (**C**) Relative expression levels of MSC-Mito DNA in the cells detected using qPCR. (**D**) In vitro assessment of the impact of MSC-Mito in conjunction with PQQ on ATP production. (**E-G**) In vitro assessment of the effect of MSC-Mito in conjunction with PQQ on ROS, SOD, and MDA levels. Scale bar: 200 μm. Values are the means ± SEMs. *P < 0.05, **P < 0.01, ***P < 0.001

Drug concentrations and treatment durations were determined based on the CCK-8 results. A concentration of 30 µM PM was used for 24 h to establish the in vitro POI model, while subsequent experiments were conducted using 20 µM PQQ and 50 µg/mL mitochondria for 24 h (Fig. S2A-C). We extracted mitochondria from human mesenchymal stem cells, stained them with MitoTracker reagent, and added them to the KGN cell model with or without PQQ. Subsequently, we used fluorescence microscope to observe the number of fluorescent mitochondria at 6 h, 12 h, 24 h and 48 h. The results showed a higher fluorescence signal from mitochondria in the cells treated with PQQ (Fig. 2A-B). Additionally, we assessed the human mitochondrial DNA content within CHO cells and observed that PQQ increased the quantity of human mitochondrial DNA in CHO cells (Fig. 2C). These results suggest that PQQ promotes the proliferation of MSC-Mito in cells.

We evaluated the impact of MSC-Mito and PQQ on mitochondrial function in KGN cells and found that the combined treatment significantly restored the mitochondrial membrane potential (Fig. S2D) and enhanced ATP production (Fig. 2D), thereby rejuvenating mitochondrial function. Furthermore, we observed that treatment with PQQ and MSC-Mito significantly reduced the elevated levels of ROS and MDA in KGN cells while increasing the content of SOD. This intervention effectively suppressed oxidative stress, and the combined treatment demonstrated superior efficacy compared to individual therapies (Fig. 2E-G). We also examined the impact of PQQ and MSC-Mito on the secretion of hormones in KGN cells. MSC-Mito and PQQ notably restored the secretion of AMH hormone compared to that in the PM group. However, individual treatments had no effect on E2 levels, whereas the combination therapy significantly increased E2 levels, approaching normal values. PQQ treatment had no effect on FSH hormone secretion, but MSC-Mito treatment and the combined therapy significantly reduced FSH levels (Fig. S2E-G).

### MSC-Mito and PQQ synergistically promote mitochondrial biogenesis while inhibiting DNA damage and cellular apoptosis

**Fig. 3:**
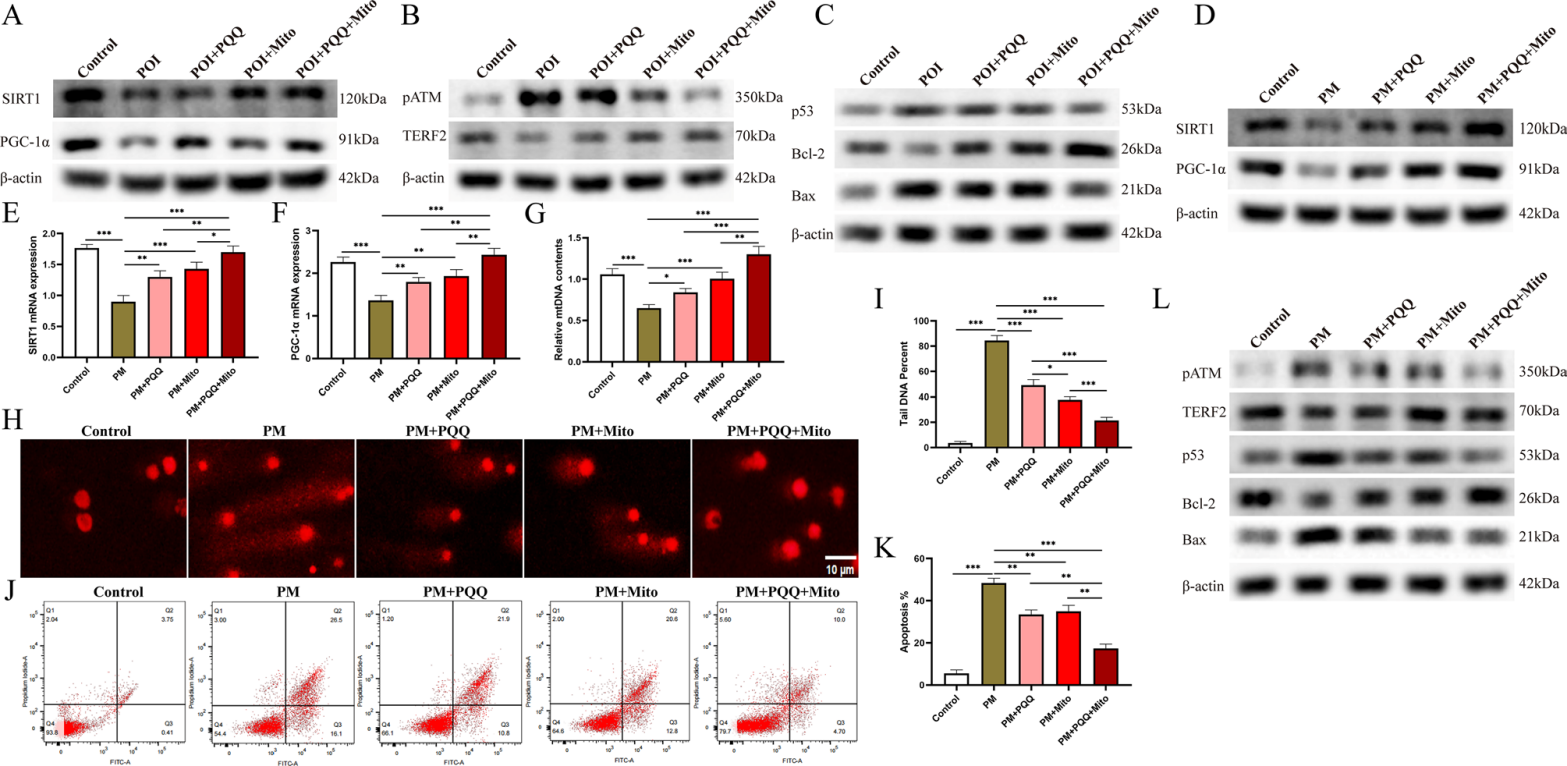
MSC-Mito and PQQ synergistically promote mitochondrial biogenesis while inhibiting DNA damage and cellular apoptosis. (**A**) Western blot analysis of SIRT1 and PGC-1α protein expression in mouse ovarian tissues of various groups. (**B**) Western blot analysis of pATM and TERF2 protein expression in mouse ovarian tissues of various groups. (**C**) Western blot analysis of the apoptosis-related proteins p53, Bax, and Bcl-2 expression in mouse ovarian tissues of various groups. (**D**) Western blot analysis of SIRT1 and PGC-1α protein expression in KGN cells. (**E-F**) qPCR analysis of SIRT1 and PGC-1α gene expression. (**G**) qPCR analysis of mtDNA copy number. (**H-I**) Comet assay to assess DNA damage. (**J-K**) Flow cytometry analysis to detect cellular apoptosis. (**L**) Western blot analysis of the impact of PQQ and MSC-Mito on the ATM/p53 signalling pathway in vitro. Scale bars: 10 μm. Values are the means ± SEMs. *P < 0.05, **P < 0.01, ***P < 0.001

SIRT1 and PGC-1α proteins play pivotal roles in antiageing processing and the promotion of mitochondrial biogenesis. We observed that the combined treatment of MSC-Mito and PQQ significantly elevated the protein expression of SIRT1 and PGC-1α in mice with POI, thereby promoting mitochondrial biogenesis (Fig. 3A). DNA damage activates the ATM signalling pathway, upregulates TERF2 protein expression, initiates DNA repair, and when DNA repair is unfeasible, activates the p53 protein to induce cell apoptosis. We found that the combined treatment of MSC-Mito and PQQ significantly reduced the phosphorylation of the ATM protein, upregulated TERF2 protein expression to facilitate DNA repair, and decreased the expression of p53 and Bax proteins while increasing Bcl-2 protein expression. These findings indicate that the combined treatment of MSC-Mito and PQQ inhibits DNA damage and reduces cell apoptosis induced by DNA damage (Fig. 3B-C). The immunohistochemistry results revealed that in mice with POI, there was a significant presence of pATM, p53, and Bax proteins within the follicle positions. Conversely, the levels of the SIRT1, PGC-1α, TERF2, and Bcl-2 proteins were notably reduced. Following treatment with MSC-Mito and PQQ, these conditions markedly improved (Fig. S3E-F).

In our in vitro experiments, we assessed the impact of PQQ in combination with MSC-Mito on mitochondrial biogenesis, DNA damage, and apoptosis. We observed that MSC-Mito, in conjunction with PQQ, significantly elevated the gene expression and protein levels of SIRT1 and PGC-1α in KGN cells. Additionally, this treatment increased the mtDNA content, promoting mitochondrial biogenesis (Fig. 3D-G). We employed the comet assay to assess the extent of DNA damage by measuring parameters such as the percentage of DNA in the comet tail, tail length, and tail moment. In the control group, comets exhibited minimal tailing, while the PM group showed a significantly higher percentage of DNA in the comet tail and longer tail length, indicating severe DNA damage. However, treatment with MSC-Mito in combination with PQQ effectively reduced the length of comet tails and decreased the percentage of DNA in the tail (Fig. 3H-I). Flow cytometry analysis was employed to assess cellular apoptosis, and we observed that MSC-Mito in combination with PQQ significantly reduced the number of apoptotic cells (Fig. 3J-K). Western blot results indicated that MSC-Mito and PQQ inhibited the expression of pATM, p53, and Bax proteins while increasing the levels of TERF2 and Bcl-2 proteins (Fig. 3L). These findings suggest that MSC-Mito, in conjunction with PQQ, can suppress the activation of the ATM/p53 apoptosis pathway, reducing DNA damage and cellular apoptosis.

### MSC-Mito coadministration of PQQ ameliorates chemotherapy-induced POI through the SIRT1/ATM/p53 pathway

**Fig. 4:**
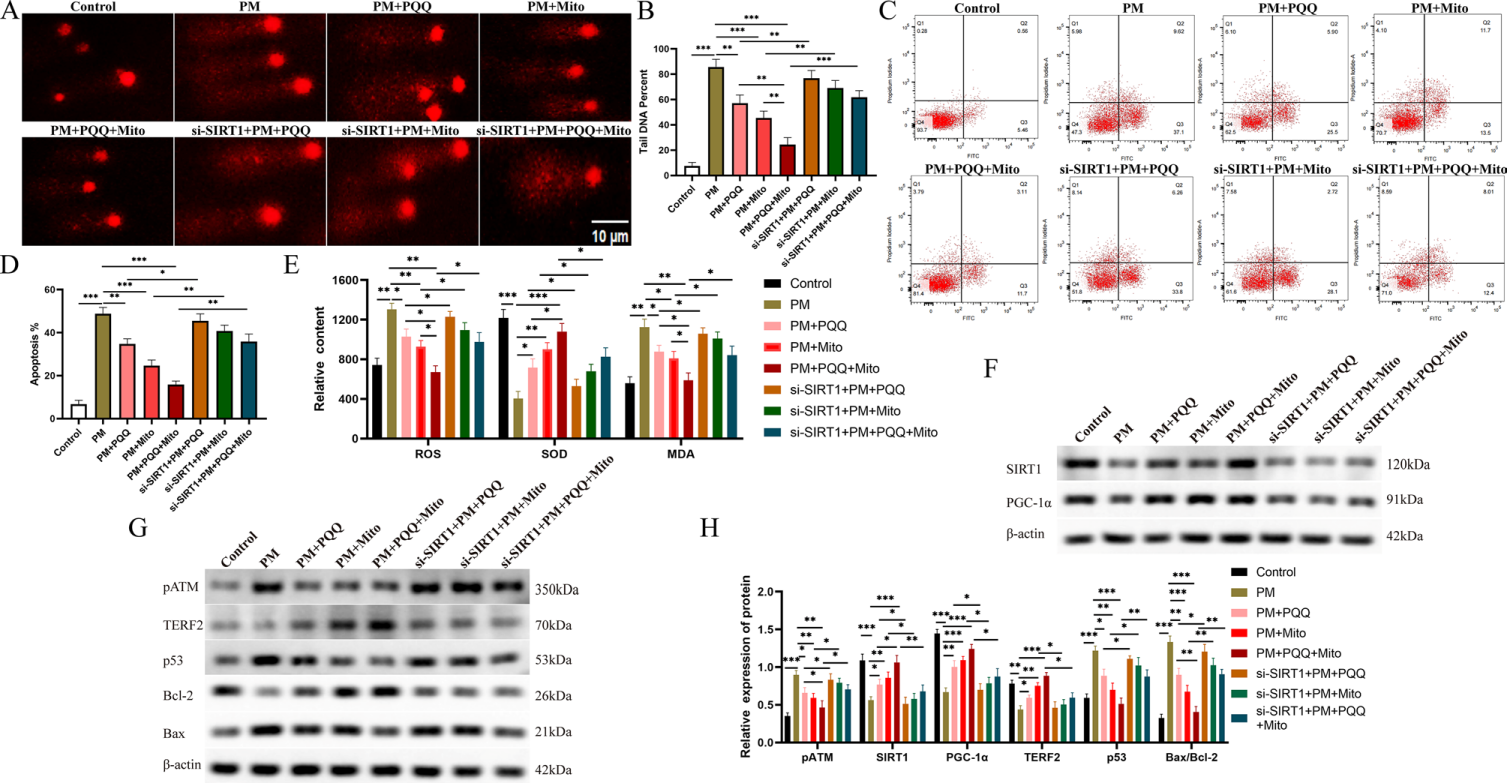
MSC-Mito coadministration of PQQ ameliorates chemotherapy-induced POI through the SIRT1/ATM/p53 pathway. (**A-B**) Comet assays were employed to assess the impact of SIRT1 knockdown on the DNA damage induced by PQQ and MSC-Mito. (**C-D**) Flow cytometry was utilized to evaluate the influence of SIRT1 knockdown on apoptosis induced by PQQ and MSC-Mito. (**E**) The effects of SIRT1 knockdown on oxidative stress induced by PQQ and MSC-Mito were examined. (**F**) Western blot analysis was conducted to investigate the influence of SIRT1 knockdown on mitochondrial biogenesis induced by PQQ and MSC-Mito. (**G**) Western blotting was employed to assess the impact of SIRT1 knockdown on the ATM/p53 signalling pathway induced by PQQ and MSC-Mito. (**H**) Quantitative analysis of Western blot results. Scale bar: 10 μm. Values are the means ± SEMs. *P < 0.05, **P < 0.01, ***P < 0.001

To investigate whether SIRT1 exerts its influence within the MSC-Mito and pyrroloquinoline quinone-mediated inhibition of the ATM/p53 signalling pathway, we employed siRNA to silence SIRT1. Our observations revealed that transfection of SIRT1 siRNA into KGN cells effectively reversed the suppressive effects of MSC-Mito and PQQ on DNA damage, concomitantly augmenting both the cellular apoptosis rate and oxidative stress levels (Fig. 4A-E). Transfection of SIRT1 siRNA diminished the expression of PGC-1α protein induced by MSC-Mito and PQQ, thereby inhibiting their facilitative role in mitochondrial biogenesis (Fig. 4F). Moreover, SIRT1 siRNA transfection suppressed TERF2 protein expression, enhanced ATM phosphorylation, and upregulated the expression of p53 and Bax/Bcl-2, effectively reversing the inhibitory effects of MSC-Mito and PQQ on the ATM/p53 signalling pathway (Fig. 4G-H). These findings indicate that MSC-Mito and PQQ suppress the activation of the ATM/p53 signalling pathway by up-regulating SIRT1 gene expression, thereby ameliorating chemotherapy-induced ovarian injury.

## Discussion

In this study, we discovered that PQQ combined with MSC-Mito regulates oxidative stress, DNA damage and apoptosis in POI model through SIRT1/ATM/p53 signal pathway, thus protecting mouse ovaries from chemotherapy-induced functional damage and premature aging. And the effect of combination therapy is stronger than that of single therapy, which may be related to the fact that PQQ promotes the proliferation of MSC-Mito in cells, thus enhancing the therapeutic effect of MSC-Mito.

Research indicates that the ovaries are particularly sensitive to cyclophosphamide, and its high gonadotoxicity leads to severe ovarian damage, including ovarian volume reduction, a significant decrease in follicles at various stages, irregular oestrous cycles, and hormonal imbalance [10, 32–34]. Curcumin, resveratrol, mesenchymal stem cells, and quercetin, among various other compounds, can exert therapeutic effects by mitigating follicular loss and augmenting ovarian reserve [35–38]. Reports indicate that PQQ may reduce follicular loss in chemotherapy-treated mice [19]. Furthermore, our earlier studies have demonstrated that liver-derived mitochondria can ameliorate the reduction in follicle numbers induced by chemotherapy. Hence, we employed a combination of PQQ and MSC-Mito to investigate whether it can restore ovarian reserve in mice with POI. We observed that the combined application of MSC-Mito in conjunction with PQQ significantly mitigates the ovarian volume and organ index reduction induced by chemotherapy, concurrently augmenting the abundance of primordial, primary, and mature follicles while reducing the number of atretic follicles. Furthermore, this treatment leads to the restoration of oestrous cycling and the secretion of AMH, E2, and FSH hormones, indicating that this combined therapy can restore ovarian function damage induced by chemotherapy, and the combination therapy has more advantages.

Studies have shown that the efficacy of standalone mitochondrial transplantation is limited, and researchers have attempted to enhance mitochondrial transfer efficiency using peptides as carriers; however, these peptides have not yet been approved for clinical use [39, 40]. Therefore, it is necessary to find other methods that can improve the efficiency of mitochondrial transplantation or enhance the therapeutic effect of transplanted mitochondria. Our research has revealed that pretreatment with PQQ can facilitates MSC-Mito proliferation to a certain extent. By employing fluorescence microscopy, we carefully observed the number of fluorescent labeled MSC-Mito in cells at different time points. Additionally, we assessed the MSC-Mito DNA content within the cells. Notably, in cells subjected to prior PQQ preconditioning, both the quantity of fluorescently labelled MSC-Mito and the amount of MSC-Mito DNA were markedly greater than those in cells not subjected to PQQ treatment.

ROS induction precipitates apoptosis in ovarian granulosa cells and the atresia of ovarian follicles, with elevated ROS levels constituting another pivotal aetiological factor underlying cyclophosphamide-induced POI [41–43]. Research indicates that metformin can mitigate granulosa cell apoptosis by inhibiting the transmission of reactive ROS signals [44]. Recent investigations have highlighted pyrroloquinoline quinone’s capacity to enhance the antioxidative potential of chemotherapy-treated murine ovaries [20]. Additionally, studies have shown that mitochondrial transplantation, as an alternative to damaged mitochondria, can ameliorate brain ischaemia‒reperfusion injuries and drug-induced renal toxicity by reducing oxidative damage [45, 46]. In the course of this study, we observed that the combined administration of MSC-Mito and PQQ substantially reduced MDA and ROS levels while augmenting the SOD content. Notably, this approach markedly suppressed the high-level oxidative stress induced by cyclophosphamide and mitigated the diminishment of ovarian granulosa cells. Furthermore, an intense inflammatory response is a contributory factor in cyclophosphamide-induced ovarian damage. Reports suggest that inflammation may exert negative effects on ovarian folliculogenesis and ovulation, thus fostering the onset of POI [47, 48]. Research by Peng Ying et al. demonstrated that berberine can ameliorate ovarian damage induced by cyclophosphamide and busulfan by reducing the inflammatory response [49]. We assessed the gene expression of the inflammatory factors IL1α, IL1β, IL6, and TNFα in mouse ovaries. Consistent with our expectations, the combined treatment with MSC-Mito and PQQ substantially diminished the gene expression of these proinflammatory cytokines, thereby suppressing the inflammatory response.

Research indicates that one of the pivotal mechanisms underlying cyclophosphamide-induced premature ovarian ageing involves the acceleration of ovarian follicular apoptosis by inducing DNA damage in oocytes and granulosa cells, thereby promoting ovarian reserve depletion [50, 51]. In vitro experiments conducted using comet assays and flow cytometry substantiated that the combined treatment of MSC-Mito and PQQ is efficacious in mitigating DNA damage and cellular apoptosis. DNA damage activates the p53 apoptosis signalling pathway, leading to cell apoptosis. In contrast to the results of the control group, our findings revealed that the ATM/p53 apoptosis signalling pathway in the POI model group was activated both in vitro and in vivo, as evidenced by elevated expression of the proapoptotic proteins p53 and Bax and a concomitant reduction in the antiapoptotic protein Bcl-2. The combined treatment of MSC-Mito and PQQ significantly diminished the phosphorylation of ATM protein, thereby suppressing the p53 apoptosis pathway. This modulation may be associated with the upregulation of TERF2 protein expression. TERF2 serves as a vital constituent of the shelterin protein complex, playing a pivotal role in maintaining telomere stability, preserving DNA structural integrity, and facilitating DNA damage repair. SIRT1, a pivotal antiageing protein in cells, regulates mitochondrial biogenesis and inflammatory responses, holding a close association with ovarian reserve and potentially serving as a marker for ovarian ageing [52, 53]. Research has indicated that the inhibition or knockdown of SIRT1 can intensify oxidative stress and DNA damage, leading to mitochondrial dysfunction and ultimately culminating in ovarian ageing and infertility [54, 55]. In this study, we observed that the combined administration of MSC-Mito and PQQ significantly ameliorates the inhibition of SIRT1 by cyclophosphamide, thereby promoting the gene and protein expression of SIRT1 and enhancing the gene and protein expression of PGC-1α, which facilitates mitochondrial biogenesis. Prior research has indicated that the SIRT1/ATM/p53 pathway can regulate cadmium-induced cellular ageing [56]. To further investigate whether the SIRT1/ATM/p53 pathway plays a role in the therapeutic effects of MSC-Mito in conjunction with PQQ for POI, we employed siRNA to silence SIRT1. Subsequently, silencing SIRT1 was found to reverse the inhibition of the ATM/p53 pathway by MSC-Mito in conjunction with PQQ. Additionally, we noted a reduction in the expression of PGC-1α and TERF2 proteins, which hindered DNA damage repair and mitochondrial biogenesis. Furthermore, the knockdown of SIRT1 resulted in elevated levels of DNA damage, cellular apoptosis, and oxidative stress. These findings suggest that MSC-Mito and PQQ may exert their therapeutic effects on POI through the SIRT1/ATM/p53 pathway.

This study has its limitations. We employed only one chemotherapy model to assess the short-term therapeutic efficacy of MSC-Mito therapy in conjunction with PQQ on POI ovarian function. Therefore, the results do not include the long-term effects of this treatment. Given the diversity of factors contributing to POI pathogenesis, further research is warranted to investigate the treatment efficacy of MSC-Mito therapy in combination with PQQ for POI induced by other factors and explore its long-term effectiveness.

## Conclusions

**Fig. 5:**
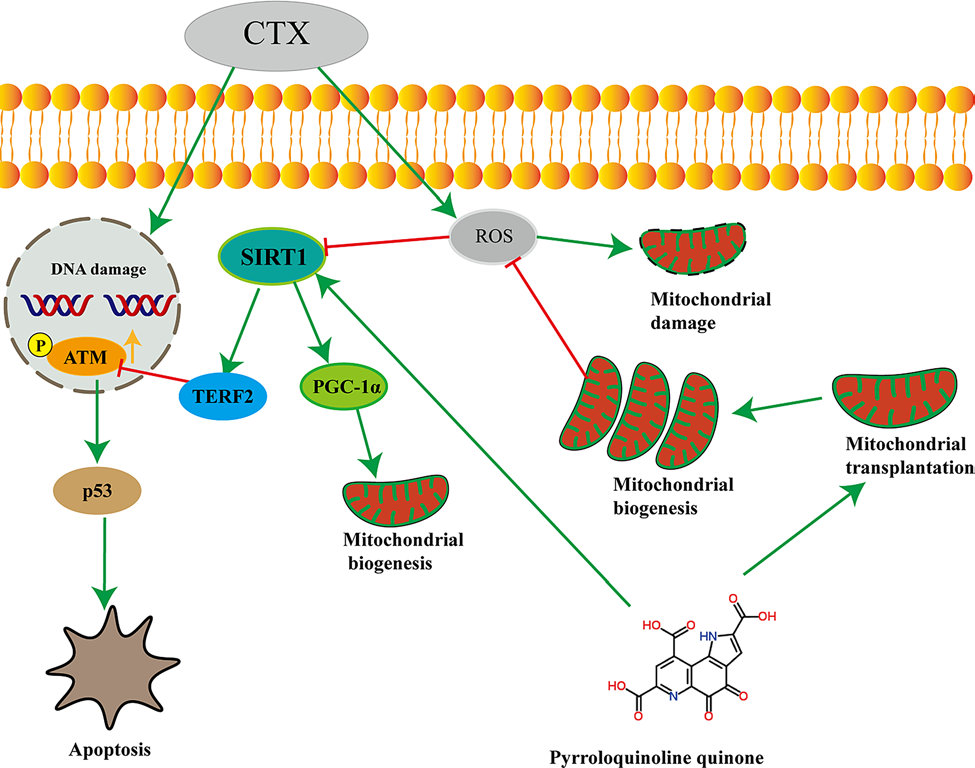
The mechanism of action for MSC-Mito therapy in conjunction with PQQ in chemotherapy-induced POI is as follows: PQQ facilitates MSC-Mito proliferation in cells and, in synergy with MSC-Mito, mitigates the elevated ROS levels induced by chemotherapy, thereby reducing mitochondrial damage. Simultaneously, this enhances the expression of SIRT1, upregulates TERF2 to promote DNA damage repair, suppresses ATM/p53 pathway activation, and reduces cellular apoptosis. Furthermore, SIRT1 upregulates PGC-1α, promoting mitochondrial biogenesis and maintaining cellular homeostasis

The outcomes of this study indicate that PQQ synergizes with MSC-Mito therapy and ameliorates chemotherapy-induced ovarian functional impairment by modulating the SIRT1/ATM/p53 pathway. This phenomenon might be associated with the facilitates MSC-Mito proliferation by pyrroloquinoline quinone(Fig. 5). These findings provide novel insights and approaches for the treatment of premature ovarian insufficiency and demonstrate the effectiveness of combined treatment with PQQ and MSC-Mito in the context of chemotherapy-induced POI.

### Electronic supplementary material

Below is the link to the electronic supplementary material.

Supplementary Material 1

Supplementary Material 2

## Data Availability

The datasets used and/or analysed during the current study are available from the corresponding author on reasonable request.

## Abbreviations

POI: Premature ovarian insufficiency
PQQ: Pyrroloquinoline quinone
ELISA: Enzyme-linked immunosorbent assays
SIRT1: Sirtuin 1
PGC-1α: peroxisome proliferator-activated receptor γ coactivator 1-alpha
ATM: Ataxia telangiectasia mutated
siRNA: small interfering RNA
E2: Estradiol
FSH: Follicle-stimulating hormone
PM: Phosphoramide mustard cyclohexanamine
ROS: Reactive oxygen species
NAD: Nicotinamide adenine dinucleotide
FOXO1: Forkhead box O1
JC-1: 5,5’,6,6’-Tetrachloro-1,1’,3,3’-tetraethylbenzimidazol-carbocyanine iodide
PMSG: Pregnant mare serum gonadotropin
AMH: Anti-Müllerian hormone
HE: Hematoxylin and eosin
IHC: Immunohistochemistry
DAB: 3,3’-diaminobenzidine
DMEM/F12: Dulbecco’s modified eagle medium/nutrient mixture F-12
RT‒qPCR: Real-time fluorescence quantitative PCR
GAPDH: Glyceraldehyde phosphate dehydrogenase
DCFH-DA: 2′,7′-Dichlorofluorescin diacetate
CCK-8: Cell-Counting-Kit-8
SOD: Superoxide dismutase
MDA: Malondialdehyde
PBS: Phosphate-buffered saline
FITC: Fluorescein isothiocyanate
PI: Propidium iodide
RIPA: Radio immunoprecipitation assay
BCA: Bicinchoninic acid assay
SDS-PAGE: Sodium dodecyl sulfate polyacrylamide gel electrophoresis
PVDF: Polyvinylidene Fluoride
TERF2: Telomeric repeat binding factor 2
Bcl-2: B-cell lymphoma-2
Bax: Bcl-2-associated X protein
ECL: Enhanced chemiluminescence
CCCP: Carbonyl cyanide 3-chlorophenylhydrazone
ATP: Adenosine triphosphate
FSHR: Follicle stimulating hormone receptor
IL1α: Interleukin-1 alpha
IL1β: Interleukin-1 beta
IL6: Interleukin- 6
TNFα: Tumor necrosis factor
mtDNA: Mitochondrial DNA
