# Correction: Pyrroloquinoline quinone promotes human mesenchymal stem cell-derived mitochondria to improve premature ovarian insufficiency in mice through the SIRT1/ATM/p53 pathway

**DOI:** 10.1186/s13287-024-03841-x

**Published:** 2024-07-11

**Authors:** Shengjie Liu, Yuanmei Wang, Hanlin Yang, Jun Tan, Jingkaiwen Zhang, Dan Zi

**Affiliations:** 1 GuiZhou University Medical College, Guiyang, Guizhou Province 550025 China; 2 Department of Gynaecology and Obstetrics, The Affiliated Hospital of Guizhou Medical University, Guizhou Medical University, Guiyang, 550004 China; 3 Department of Gynecology, Guizhou Provincial People’s Hospital, Guiyang, Guizhou 550025 China; 4 Key Laboratory of Endemic and Ethnic Diseases and Key Laboratory of Molecular Biology, Ministry of Education, Guizhou Medical University, Guiyang, 550004 China; 5 College of Clinical Medicine, Guizhou Medical University, Guiyang, China; 6 Department of Gynecology and Obstetrics, The Afliated People’s Hospital of Guizhou Medical University, Guiyang, China

**Correction: Stem Cell Research & Therapy (2024) 15:97**

10.1186/s13287-024-03705-4

The original article erroneously omits an affiliation for co-author, Dan Zi.

The correct affiliations for Dan Zi can be viewed via this correction article.

