# Supplementary material for: Pyrroloquinoline quinone promotes human mesenchymal stem cell-derived mitochondria to improve premature ovarian insufficiency in mice through the SIRT1/ATM/p53 pathway
Source: Stem Cell Res Ther. 2024 Apr 5;15:97. doi: 10.1186/s13287-024-03705-4 (PMC10998350; doi:10.1186/s13287-024-03705-4)
Supplement: Supplementary file 1 — Supplementary Material 1 [file 13287_2024_3705_MOESM1_ESM.docx]

**Supplementary Materials**


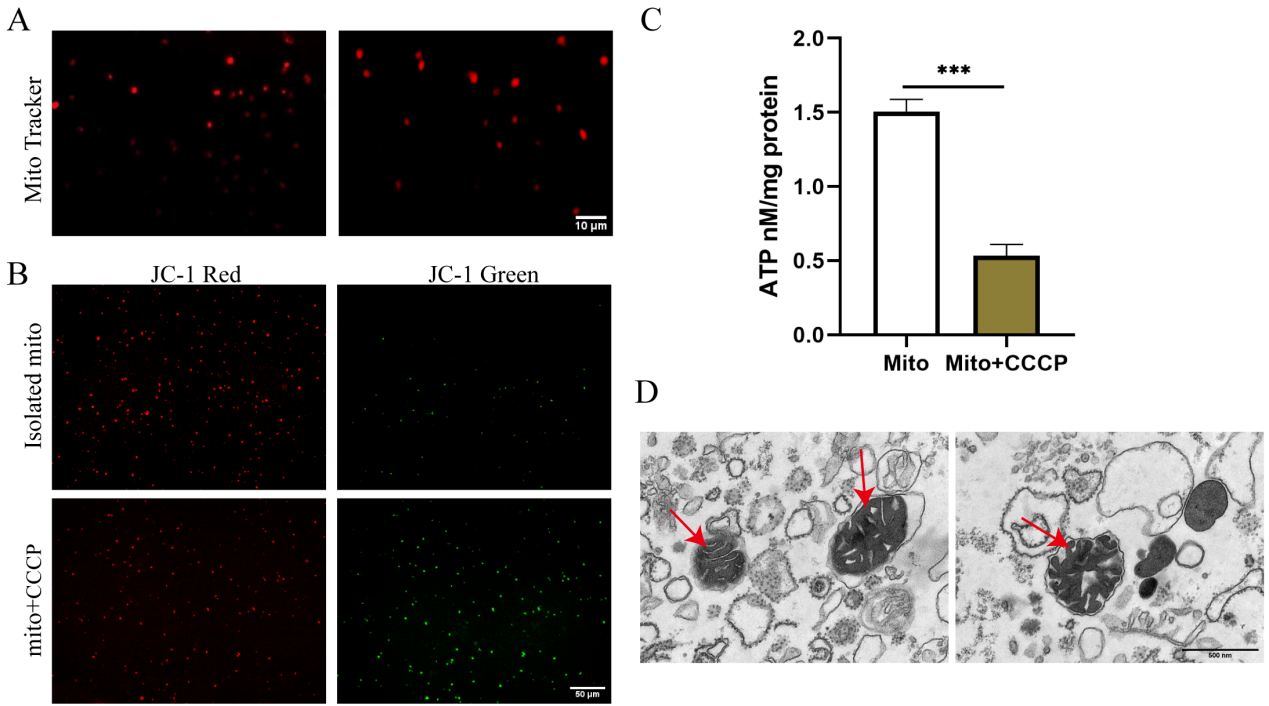


Fig S1. Functional identification of MSC-Mito. (A) Assessment of mitochondrial activity in mesenchymal stem cell sources using MitoTracker staining. Scale bar: 10µm. (B) Evaluation of mitochondrial membrane potential in mesenchymal stem cell sources using JC-1. Scale bar: 50µm. (C) Measurement of ATP production from mitochondria derived from mesenchymal stem cells. (D) Morphological examination of mitochondria derived from mesenchymal stem cells using transmission electron microscopy. Red arrows indicate mitochondria. Scale bar: 500nm. Values are the means ± SEMs. ***P < 0.001。


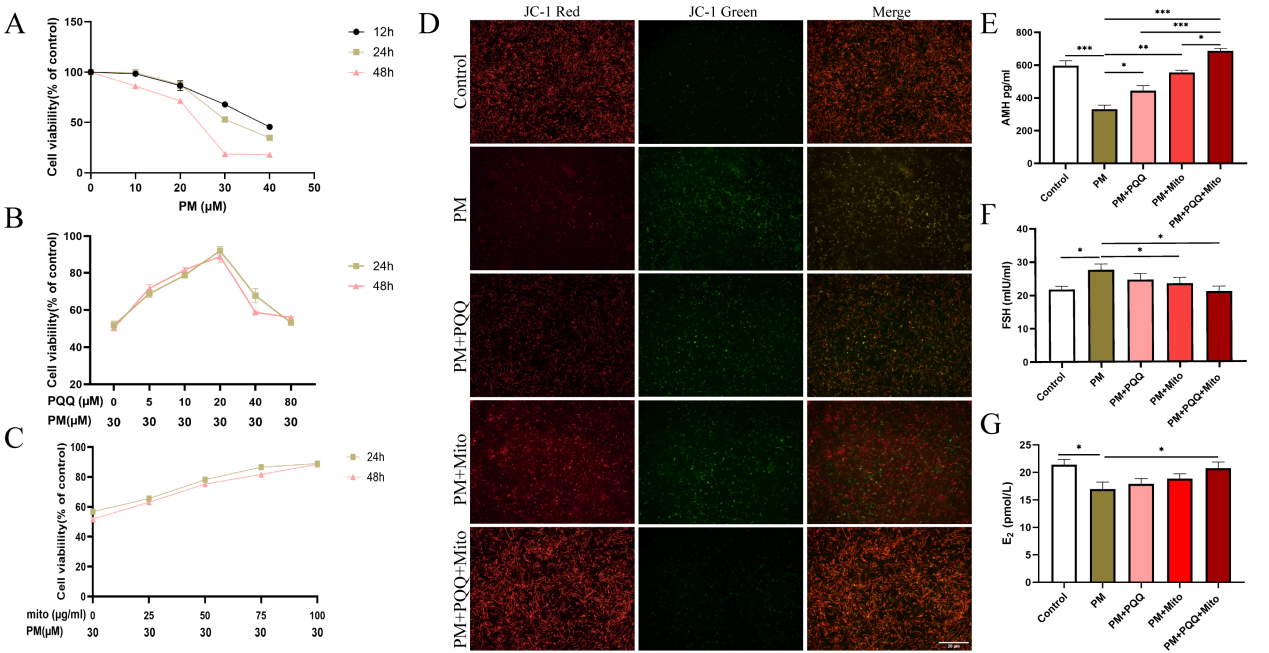


Fig S2. MSC-Mito in conjunction with PQQ restore mitochondrial membrane potential in vitro and improve hormonal secretion. (A-C) Screening of effective concentrations and exposure times of PM, PQQ, and MSC-Mito in vitro using the CCK-8 assay. (D) Evaluation of the synergistic impact of PQQ and MSC-Mito on mitochondrial membrane potential in KGN cells using JC-1. (E-G) Assessment of the combined effect of PQQ and MSC-Mito on sex hormone secretion in KGN cells using ELISA. Scale bar: 50µm. Values are the means ± SEMs. *P < 0.05, **P < 0.01, ***P < 0.001.


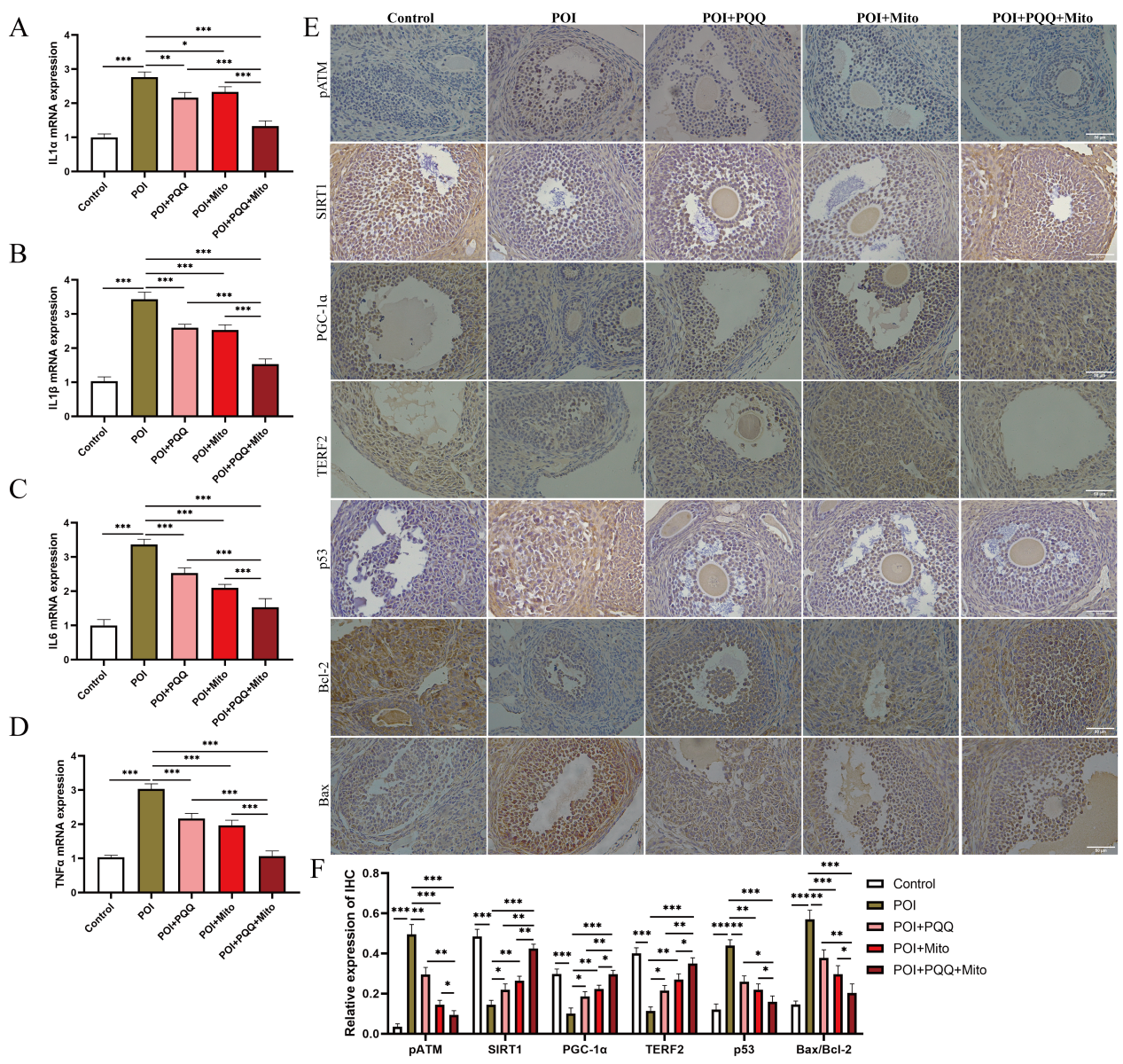


Fig S3. MSC-Mito in conjunction with PQQ promote mitochondrial biogenesis, inhibit DNA damage, cellular apoptosis, and inflammatory responses in vivo. (A-D) qPCR analysis of gene expression of inflammatory factors IL1α, IL1β, IL6, and TNFα in mouse ovarian tissues. (E-F) Immunohistochemical examination of the protein expression of SIRT1, PGC-1α,and ATM/p53 signaling pathways in mouse ovaries following treatment with PQQ in combination with MSC-Mito. Scale bar: 50µm. Values are the means ± SEMs. *P < 0.05, **P < 0.01, ***P < 0.001.
