# Supplementary figures and images for: Pyrroloquinoline quinone promotes human mesenchymal stem cell-derived mitochondria to improve premature ovarian insufficiency in mice through the SIRT1/ATM/p53 pathway
Source: Stem Cell Res Ther. 2024 Apr 5;15:97. doi: 10.1186/s13287-024-03705-4 (PMC10998350; doi:10.1186/s13287-024-03705-4)

Fig3A-B

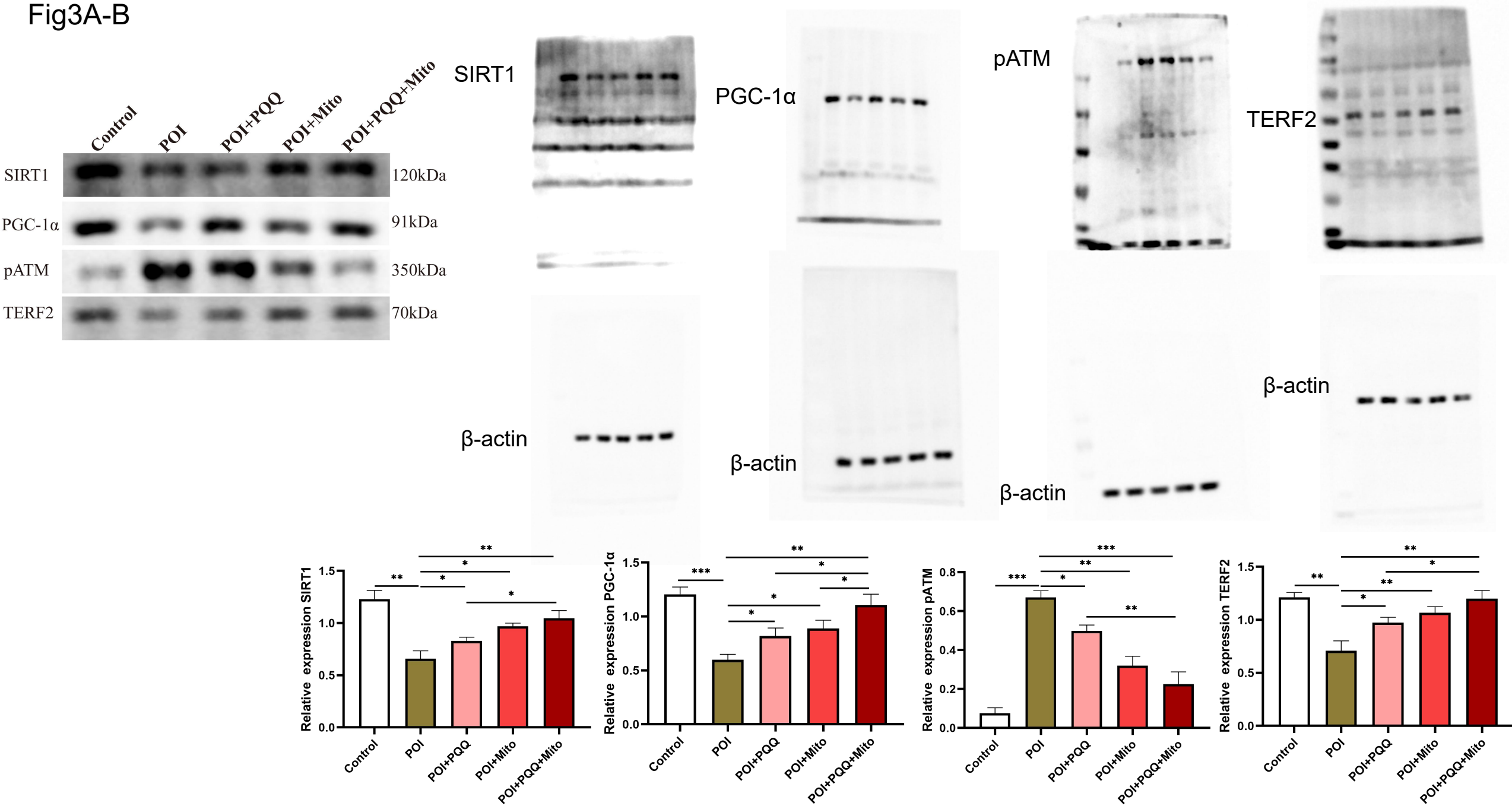

Fig3C

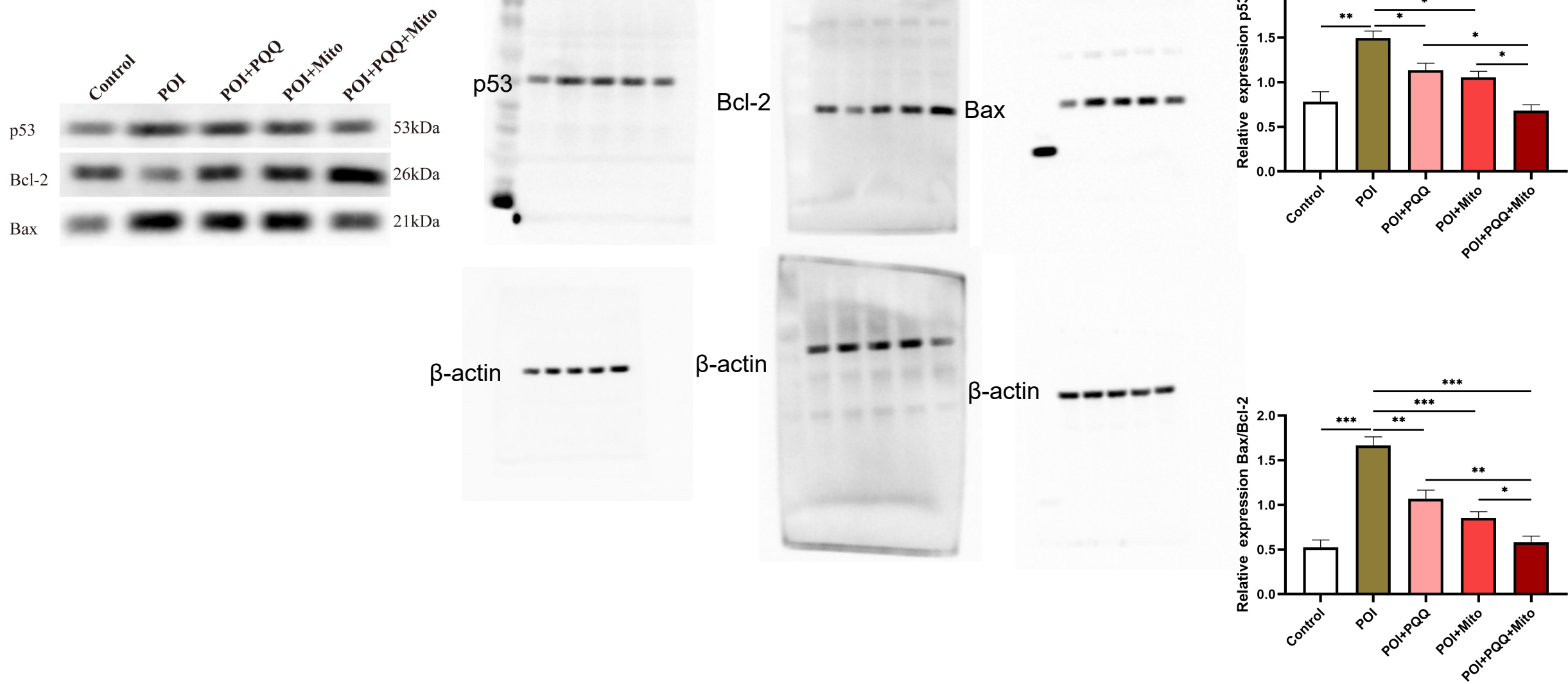

Fig3D, Fig3L

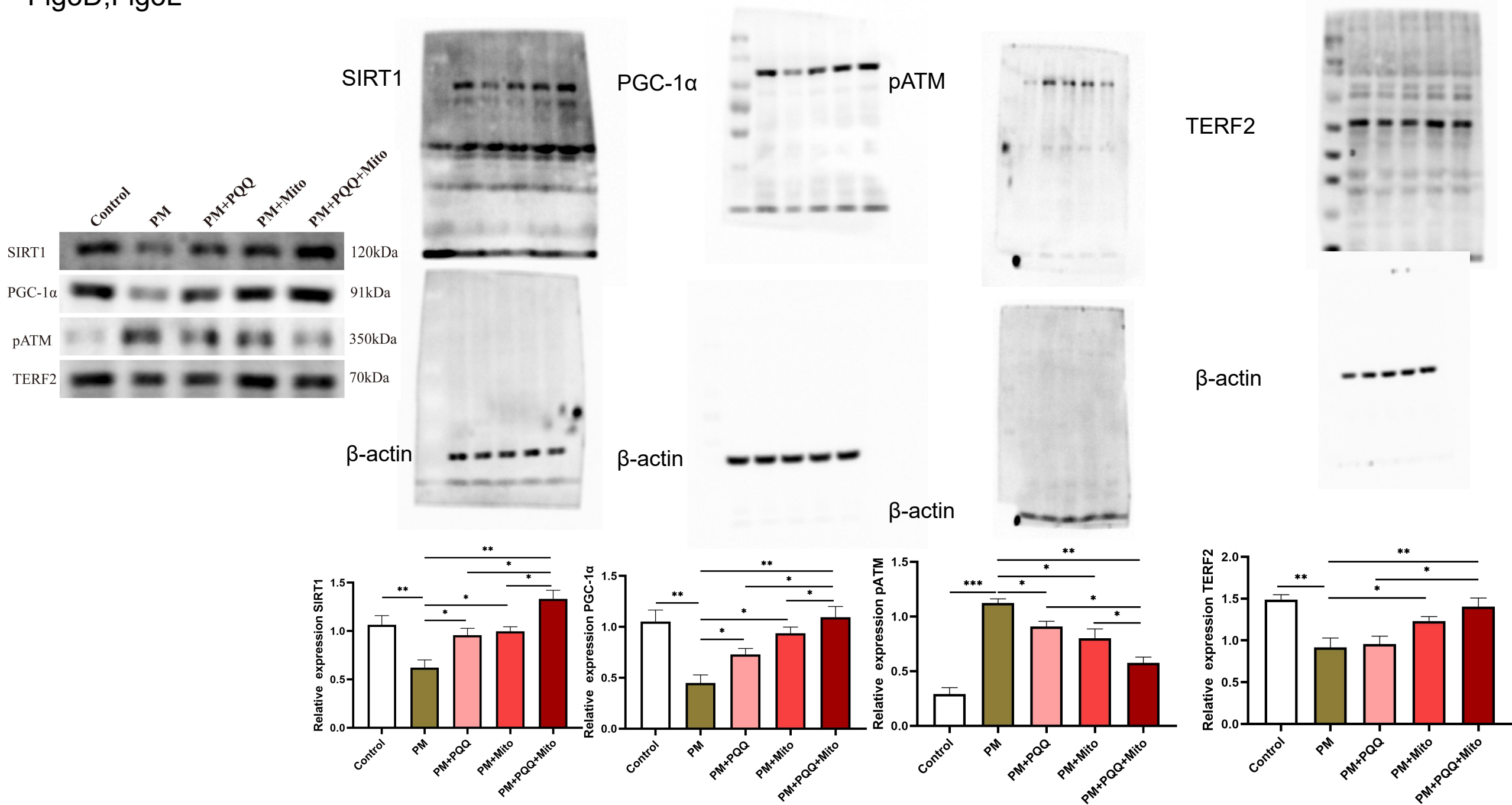

Fig3L

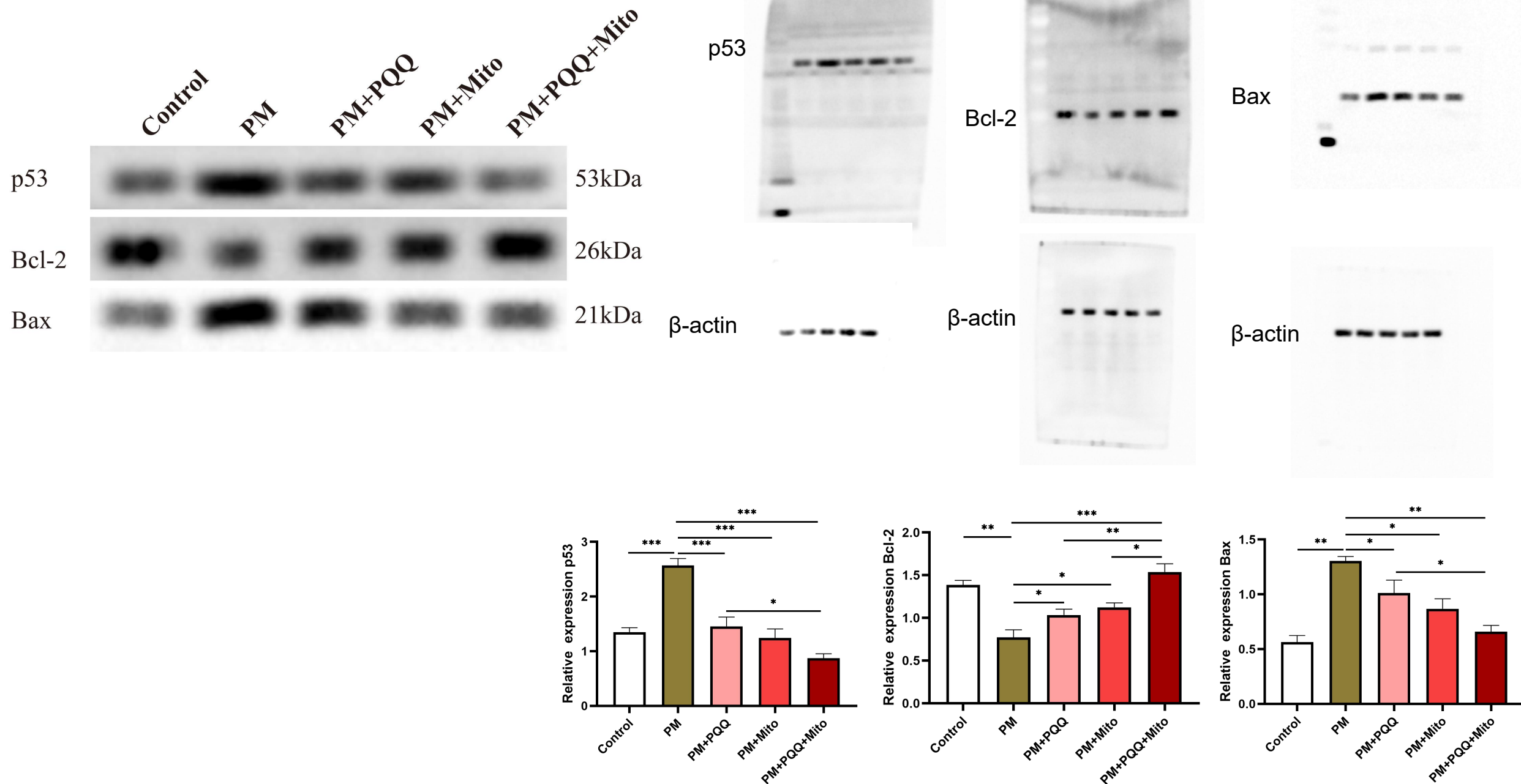

Fig4F

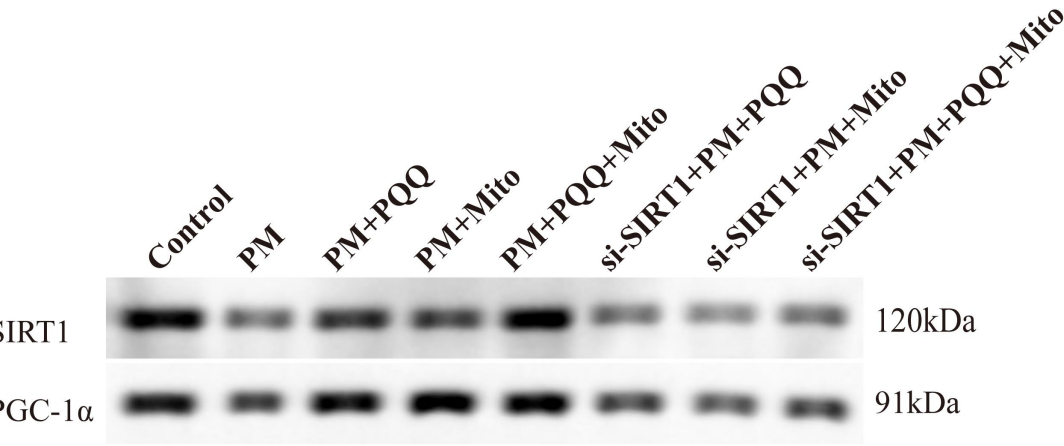

SIRT1

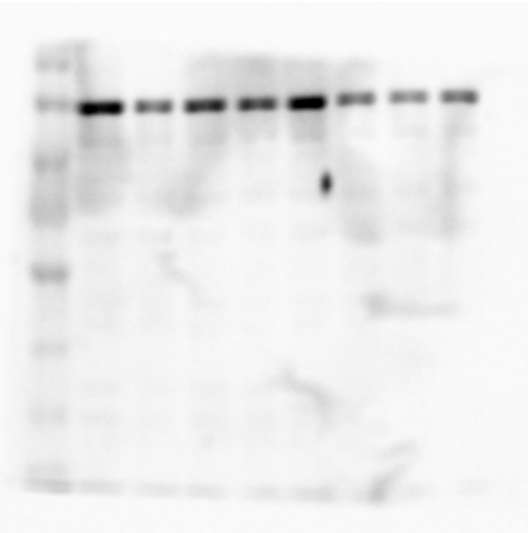

PGC-1α

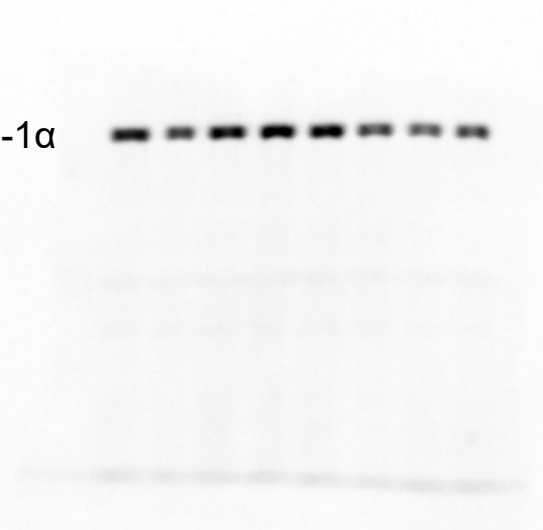

β-actin

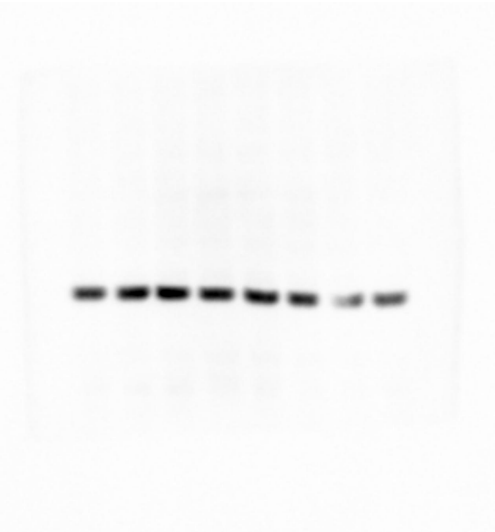

β-actin

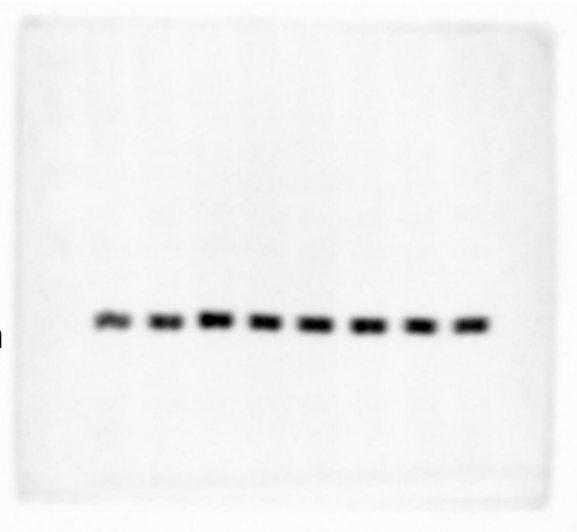

Fig4G

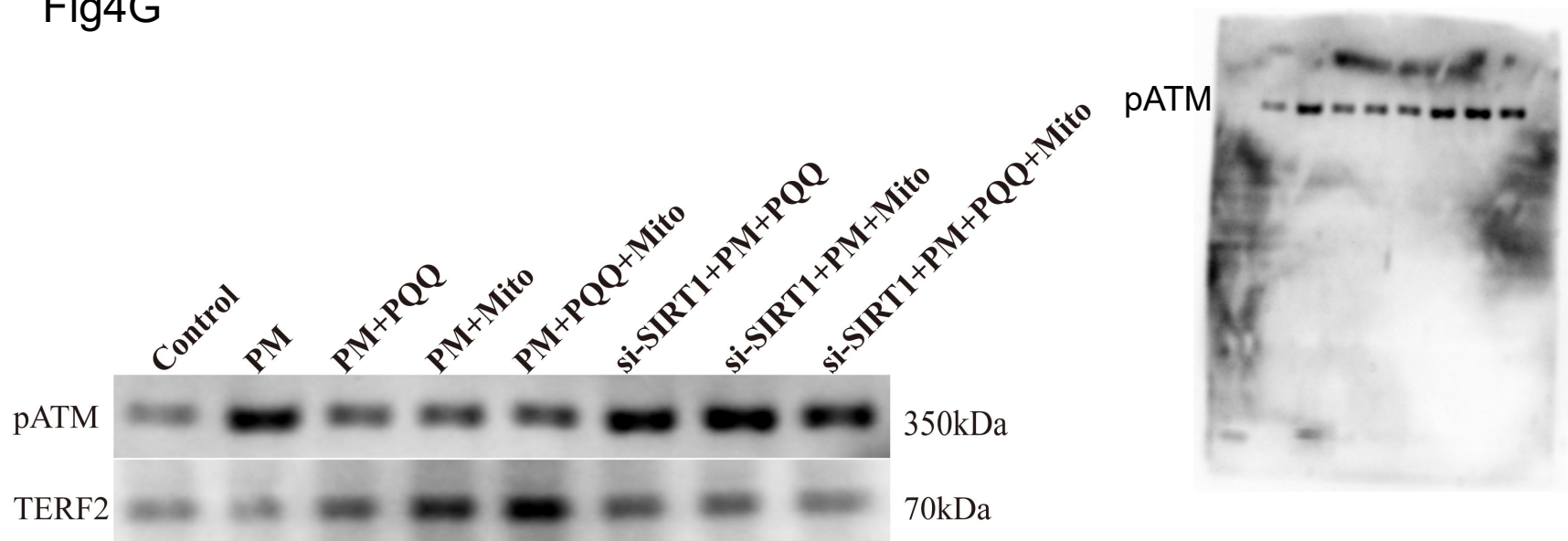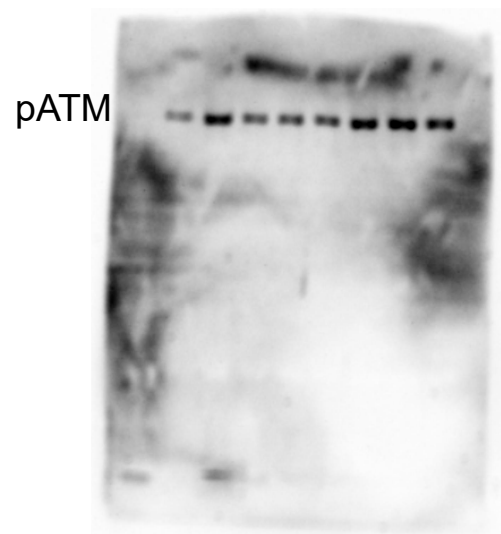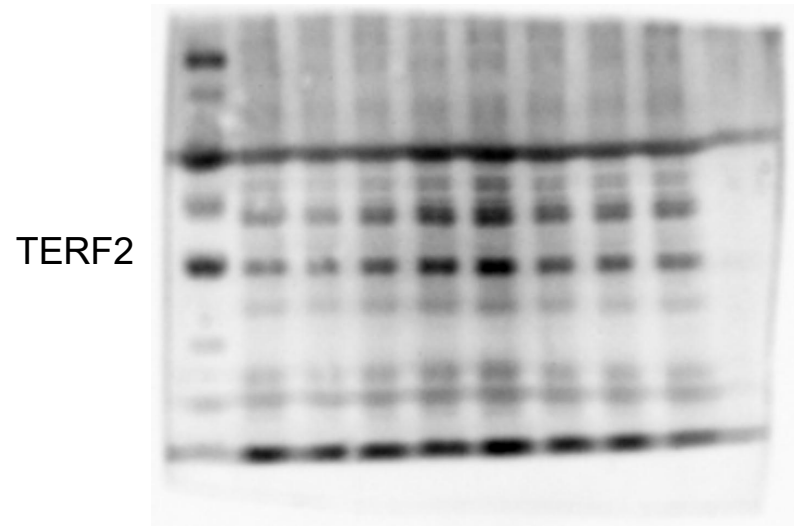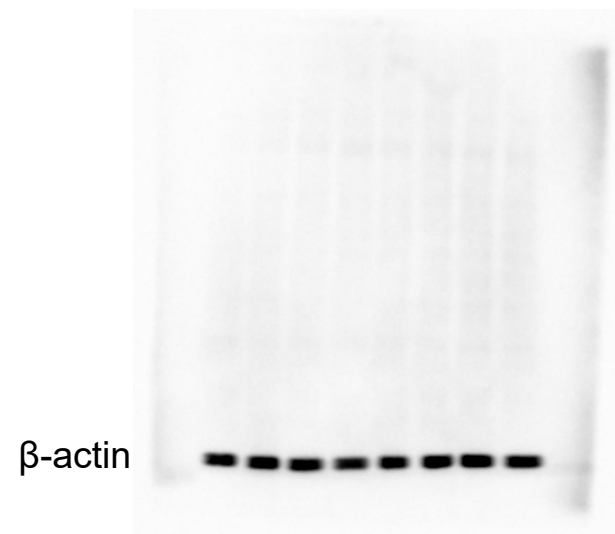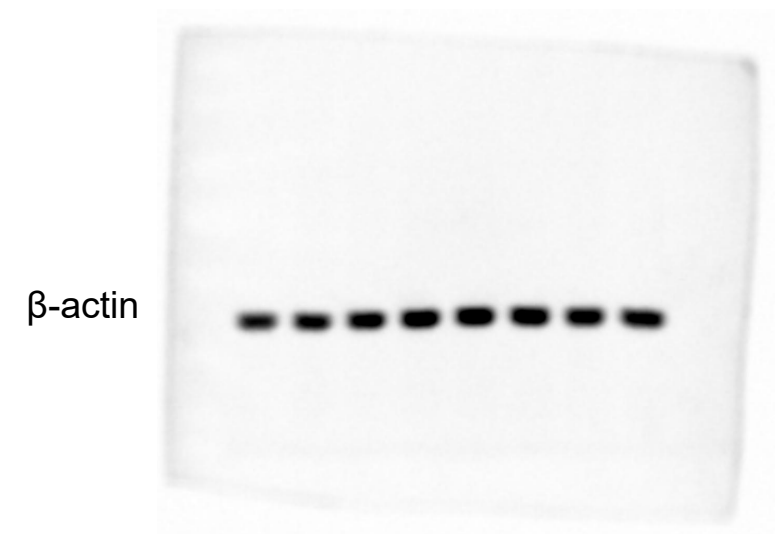

Fig4G

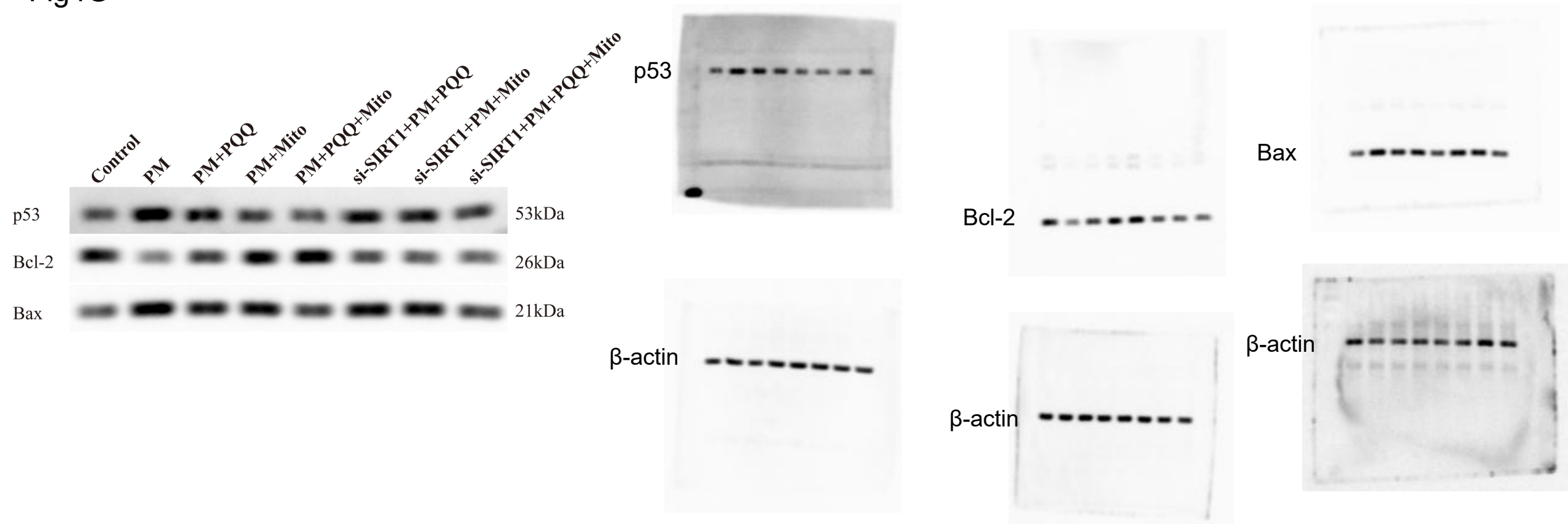

Fig4F-G

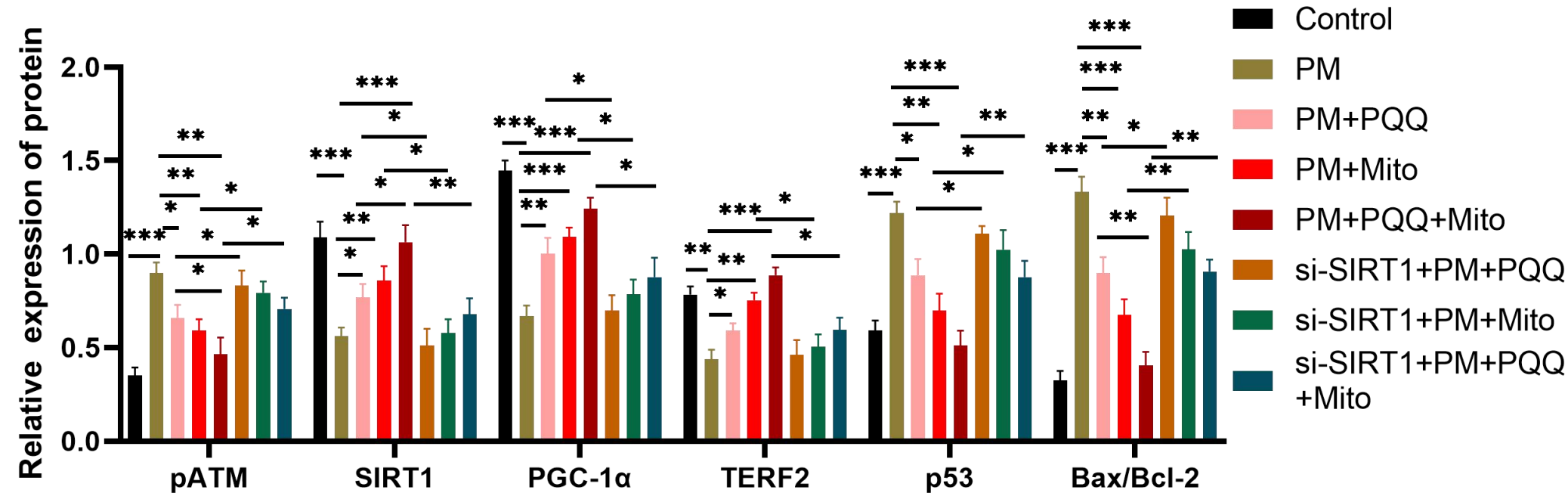

Supplement: Supplementary file 2 — Supplementary Material 2 [file 13287_2024_3705_MOESM2_ESM.pdf]
